# Stochastic dynamic programming for condition-based maintenance and production considering predetermined demand and maintenance delay in a finite planning horizon

**DOI:** 10.1371/journal.pone.0357000

**Published:** 2026-09-02

**Authors:** Pourya Mohammadipour, Hiwa Farughi, Hasan Rasay

**Affiliations:** 1 Department of Industrial Engineering, Faculty of Engineering, University of Kurdistan, Sanandaj, Iran; 2 Department of Industrial Engineering, Kermanshah University of Technology, Kermanshah, Iran; Beijing Institute of Technology, CHINA

## Abstract

Today, equipment, sensors, and the Internet of Things have enabled remote monitoring and control of production equipment in less time. These technologies can be used for condition-based maintenance to reduce costs and increase reliability. In addition to maintenance actions, another approach to controlling system failure is adjusting the production rate, which is also known as condition-based production. In this paper, both condition-based maintenance and production decisions are integrated. We study the problem of optimizing the integrated production and maintenance of a deteriorating machine, where maintenance delays are considered. A finite planning horizon that includes several periods is considered and at the beginning of each period, the state of the system will be obtained. In each period, the production system has predetermined demand and random failures occur based on the production rate. If the machine enters a failure state, corrective maintenance must be scheduled. Otherwise, the decision-maker can choose to either schedule preventive maintenance or decide on the production rate. The problem is modeled within the framework of a Markov Decision Process, where the total production and maintenance costs over a finite planning horizon are minimized. Finally, the proposed model is solved using the value iteration algorithm to determine the optimal policies. A numerical example and case study are provided to demonstrate the applicability of the model. In this study, the proposed Condition-Based Maintenance and Production policy is compared with fixed production and maintenance policies. According to the results obtained, the proposed dynamic Condition-Based Maintenance and Production policy outperforms the static policies. The problem presented for the case where the maintenance delay is zero is also modeled, and the results for the Condition-Based Maintenance and Production dynamic policy are presented. In addition, in this case, the value of the optimal initial state for different constant policies is obtained and compared. In this case, as well, the dynamic policy performs better than other static policies.

## 1. Introduction

The deterioration and failure of machines and equipment in production systems can be attributed to a range of environmental factors, including temperature, humidity, variations in energy, and challenging working conditions. These environmental factors can accelerate the deterioration and degradation of mechanical components, leading to failures if not properly monitored and maintained. For example, high temperatures can cause overheating, leading to the deformation of metal parts or failure of electronic components. Humidity can lead to corrosion, while energy fluctuations can result in electrical failures. Hard working conditions, such as continuous operation or operation under heavy loads, increase stress on equipment, further hastening their deterioration.

If these failures are not detected and repaired in time, they may lead to the total failure of the system. Such failures can have harmful effects, including safety problems for workers, incurring high costs due to emergency repairs and downtime, damaging other interconnected equipment, reducing the quality of production, and causing sudden halts in the production process. These consequences highlight the critical importance of preventive maintenance activities to maintain or restore equipment to acceptable operational conditions.

Maintenance activities Includes a variety of tasks, from minor ones like cleaning and changing oil to major ones such as replacing large components. These activities constitute a significant part of the production costs for many factories and organizations. For example, maintenance costs often account for 15–40% of total variable costs in factories [1]. Power plants and offshore wind farms, known for their intensive operational demands, spend up to 30% of their total life cycle costs on maintenance [2,3]. During the operation phase of wind turbines, maintenance activities can average 67% of the total costs, with some instances reaching more than 85% [4]. These significant costs clearly demonstrate that effective maintenance operations and strategies are crucial to the profitability and competitiveness of companies.

In maintaining production systems, it is crucial to implement effective and efficient maintenance actions and select the appropriate maintenance strategies. Traditional maintenance strategies, such as reactive maintenance, involve repairing equipment only after it has failed. While this approach might reduce short-term costs, it often results in higher long-term costs due to unplanned downtime and secondary damages. Preventive maintenance, on the other hand, involves performing regular, scheduled maintenance activities to prevent unexpected failures from occurring. However, this approach can also be inefficient, as maintenance might be performed on equipment that does not need it yet. Today, more advanced maintenance strategies are being adopted, such as condition-based maintenance (CBM). This approach involves carrying out maintenance operations based on the actual condition of the equipment, rather than on a fixed schedule. CBM relies on the monitoring of machinery conditions, which can be done continuously or at specific intervals. Sensors and diagnostic tools are used to collect data on various parameters, such as vibration, temperature, and noise levels. This data is then analyzed to detect early signs of degradation and potential failures, allowing maintenance to be performed only when necessary.

CBM is particularly useful in systems where reliability is of high importance. Since machinery failures do not typically occur suddenly but rather develop over time, CBM can provide early warning signals before an accident occurs. For example, an increase in vibration might indicate misalignment or imbalance, while a temperature rise could signal excessive friction or a failing cooling system. By monitoring these signals, maintenance can be scheduled proactively, thus preventing unexpected breakdowns and minimizing downtime.

In manufacturing systems, effective performance of maintenance activities is a key factor in maintaining productivity, reducing downtime, and increasing equipment reliability. However, in real industrial conditions, maintenance operations are often not performed immediately after a failure occurs. One of the most important reasons for this delay is the need for coordination between maintenance teams, operators, spare parts inventory, and production planning. Therefore, a faster operational option should be proposed to avoid equipment failure before maintenance operations can be carried out, thus preventing the production system from stopping. One effective method to control the failure rate is by managing the production rate.

There is a clear relationship between the failure rate of a system and its production rate. At higher production rates, equipment tends to deteriorate faster due to increased stress and operational loads. For instance, wind turbine gearboxes and generators break down more quickly when operating at high speeds, conveyor belts are more prone to failure when used at higher rotational speeds, trucks experience faster wear and tear when loaded heavily, and large supercomputers often fail under higher workloads. Cutting tools wear out faster at higher speeds, and stamping machines are more likely to break down with increasing stamping speeds. In these cases, it can be useful to adjust the production rate based on system condition information to increase or decrease production, thereby preventing possible costly failures before maintenance operations are required. This approach is known as condition-based production. By using condition information, production planners can make informed decisions to optimize the production rate according to the condition of the system. For instance, if the condition monitoring system detects that a particular machine is showing signs of degradation, the production rate can be reduced to extend the machine’s operational lifetime until maintenance can be performed. Conversely, if the system is in optimal condition, production rate can be increased to maximize output. It should be noted that sometimes in CBP it is advised to increase the production rate in order to intensify the system deterioration, for example, for clustering the maintenance activities.

CBP not only helps in preventing sudden equipment failures but also enhances the overall efficiency and productivity of the production system. By integrating maintenance strategies with production planning, companies can achieve a more balanced and sustainable operational approach, ultimately leading to improved profitability and competitiveness in the market. Furthermore, advancements in technology, such as the Internet of Things, artificial intelligence, and machine learning, are enhancing the capabilities of condition-based maintenance and production. IoT devices can provide real-time data from various parts of the production system, while AI and machine learning algorithms can analyze this data to predict failures and optimize maintenance schedules. These technologies enable more precise and timely maintenance actions, reducing downtime and extending the lifespan of equipment. By embracing these technologies, companies can further refine their maintenance and production strategies, ensuring that they remain competitive in an increasingly demanding market. In conclusion, the effective management of maintenance and production rates based on real-time condition monitoring is essential for the sustainability and profitability of modern production systems. By adopting condition-based maintenance and production strategies, companies can not only prevent costly equipment failures but also optimize their production processes, ultimately leading to a more efficient and competitive operation.

In this paper, we begin by reviewing the literature on CBM, CBP and CBMP in Section 2. This section provides a detailed classification of previous studies, highlighting key developments in each field. Following this, Section 3 introduces our proposed problem, articulating the specific maintenance and production challenges. Section 4 develops a model for the proposed problem using the Markov decision process (MDP) framework, aiming to capture the stochastic nature of the decision-making environment. To evaluate the performance of the model, Section 5 presents an example that illustrates its application and effectiveness. To illustrate the practical applicability of the proposed problem, a case study is presented in Section 6. Furthermore, Section 7 provides a comparative analysis of various static policies against the dynamic policy introduced in this study. Finally, Section 8 provides a summary and discussion of our findings, along with potential directions for future research in CBM and CBP.

## 2. Literature review

In the current literature, most of the maintenance and production decisions are considered and optimized separately. For example, Keizer et al. in 2017 considered a multi-unit parallel production system assuming the existence of failure dependence and economic dependence. In this research, the problem is formulated as an MDP model and the optimal maintenance policy is provided when the average cost is minimized in the long run [5]. In 2019, Truong-Ba et al. proposed a CBM strategy considering partial opportunities to perform maintenance operations under budget-constrained conditions. Moreover, their research includes the analysis of a set of imperfect maintenance measures. In this research, average maintenance costs are minimized through an MDP model, and system failure thresholds for different protection measures and opportunistic maintenance selection are jointly optimized [6].

In 2026 Cheikh and Boudi, investigated dynamic optimization in a condition-based maintenance framework, in which the equipment deterioration process is modeled stochastically and the performance of different maintenance policies is evaluated using Monte Carlo simulation. The results of this study show that utilizing system condition information and making dynamic decisions can significantly improve system performance in terms of reliability and maintenance costs [7].

Zhou et al. [2026] investigate the joint optimization of maintenance, spare-parts inventory, and remanufacturing decisions in multi-unit systems. The problem is formulated as a Markov decision process that accounts for the stochastic nature of equipment deterioration as well as multiple remanufacturing cycles [8]. In 2022, Yang et al. investigated the condition-based maintenance problem for redundant systems with arbitrary structures, considering both failure and economic dependence among components [9]. Wang et al. (2025) developed an integrated optimization model of quality control and maintenance for production systems subject to quality-dependent failures, addressing the common assumption in the literature that non-conforming products do not affect system degradation [10]. In another study, Wang et al. (2025) proposed a joint optimization model of maintenance and two-way (longitudinal and lateral) spare-parts transshipment policies for balanced systems, in which the failure of a unit requires its symmetric counterpart to stop working [11]. Ma et al. (2025) proposed a multi-level adaptive risk control framework that jointly optimizes routine maintenance and task-termination decisions for heterogeneous degrading systems operating under uncertainty [12].

In 2026, Mashaykh et al. presented a Bayesian framework for integrating quality control, condition-based maintenance, and production planning in multivariate processes. In this study, Bayesian control charts and multi-state failure modeling were used for system monitoring and optimization was performed using meta-heuristic methods [13].

In 2023, Park and Pham presented a CBM strategy for a failure-dependent system with two sources of failure and random shocks. In this research, a reliability model has been developed for a system under failure, where the dependence between the degradation process and random shocks is considered, and a warranty cost analysis has been performed based on the proposed model [14]. In 2024, Ward et al. used machine learning models to obtain the optimal CBM policy where condition monitoring is imperfect [15]. Rasay et al. in 2024 propose an advanced preventive maintenance model considering imperfect maintenance, where minor repairs are limited and affect the system’s failure process based on a geometric process [16].

The literature on maintenance is extensive and various types of maintenance interventions have been considered. The first differentiation that can be made is between preventive and corrective maintenance strategies. Preventive maintenance operations can be time-based or condition-based, and policies of both types have been studied and compared in research studies [17–20]. These studies show that CBM strategies reduce system operating costs and improve reliability. The studies mentioned above highlight the value of dynamic maintenance strategies, but the importance of dynamic control of system deterioration has not been studied in them.

In production planning, three primary research fields can be classified based on the relationship between the production rate and failure rate. In the first type of studies, it is assumed that there is no relationship between production rate and failure rate. For example, in this type of studies, it is assumed that system failure depends only on the age factor. In the second type, it is assumed that the production rate is related to the failure rate. But the production rate is not changed to control the failure. For example, the production rate is changed periodically to respond to demand, not to control the rate of system degradation. In the third type, it is assumed that the production rate is related to the failure rate, and the production rate is adjusted to control the degradation of the system. In the following, some of the studies conducted in all three categories are reviewed and presented. Francie et al. in 2014 examined a production system with two parallel components and constant demand. In this research, the failure rate of one machine depends on its production rate, and the failure rate of the other machine was assumed to be constant [21]. Lu et al. in 2015 sought to find a stable production plan for a single-component system with availability constraints. They assumed that the system failure depends on its age and the system will experience unexpected failures. Then they proposed a joint model for integrating preventive maintenance into the production scheduling problem, where the sequence of tasks and the maintenance times are determined simultaneously [22].

Hu et al. in 1994 showed that the system reliability can be improved by reducing the production rate [23]. Research on production systems with adjustable production rates, condition monitoring, and the relationship between failure and production rates is just a few studies. Based on a review of the literature, we can say that the relationship between production decisions and system failure behaviors has been extensively studied; however, the value of using condition monitoring to dynamically adjust the production rate and thus the failure rate has been neglected.

The first research in this field that considered the relationship between the production rate and the failure rate, and based on the information of the system conditions, presented the joint decisions of maintenance and production, was the article of Broek et al. which was carried out in 2021. They considered a single-component system and continuously monitored the system status. In this research, when the maintenance operation is planned to increase the system reliability and prevent the system from stopping, the production rate can be changed. In this research, the MDP model is solved by the value iteration algorithm, and the optimal maintenance and production policy is presented to minimize the cumulative costs of the system [24].

In another study by Broek et al. in 2021, they extended their previous paper to a system with two components [25]. Koopmans and De Jong in 2023 considered a parallel multi-component production system. In this research, it is assumed that two types of corrective and preventive maintenance will be done, considering the delay in maintenance. In the production system, it is assumed that each unit can work with three production rates (1, 0.5, 0), and as the production rate increases, the failure rate will also increase. This problem is modeled in the form of an MDP and solved by the value iteration algorithm. The optimal policy of maintenance and production is determined to maximize profit on an infinite planning horizon [26].

Li et al. (2026) develops a condition-based maintenance approach for multi-stage serial production systems that simultaneously accounts for reliability and product quality. The health condition of quality-related components together with key product characteristics is monitored jointly, using a combination of a multivariate statistical control chart and a failure-rate model to assess system status; the resulting finite-horizon optimization problem is solved by means of a simulation-based genetic algorithm [27]. In 2023, Drent et al. analyzed a single-unit production system subject to random failure under a condition-based production structure. In this article, a Bayesian learning algorithm is used to show the relationship between failure rate and production rate. In this research, first, an operational decision-making problem to balance failure and profit by using production rate adjustment is investigated and this problem is modeled with MDP. Then the optimal maintenance scheduling times are obtained using the Bang-Bang policy [28].

Fang et al. (2026) proposes a condition-based maintenance policy for mission-oriented systems subject to stochastic missions with uncertain durations. In their study, the system degradation process is modeled as being directly influenced by the type and sequence of missions, and maintenance decisions are made based on information obtained from post-mission inspections; the maintenance optimization problem is formulated as a semi-Markov decision process and solved using a policy iteration algorithm [29].

Mohammadipour et al. (2025) studied a single-component production system and proposed an integrated condition-based maintenance and condition-based production model under predetermined demand and a finite planning horizon. They showed that incorporating production decisions into maintenance policies reduces failure risk, decreases the number of maintenance actions, and minimizes total system cost using an MDP framework solved by value iteration [30].

In 2023, Sun et al presented a preventive maintenance strategy for a manufacturing system whose production rate can be dynamically adjusted before maintenance operations. The purpose of this article is to optimally control the failure rate and create a balance between the risk of failure and production profit. In this research, it is assumed that the increase in the failure rate due to the increase in the production rate during a period is uncertain [31]. In 2024, Zhang et al. investigated the integrated optimization of production and maintenance in a single-unit production system. They assumed that the failure of the system is random and is proportional to the production rate [32].

The following table summarizes past research in this field. In general, in the research conducted in this area, the time horizon has been considered either finite or infinite. While in the finite horizon problem optimizing the expected costs/profits during the planning horizon is usually taken into consideration, for the infinite counterpart, optimizing expected costs/profits per time unit is usually defined as the objective function.

Regarding the effects of maintenance actions, they can be generally classified into perfect and imperfect. In the perfect case, it is assumed that maintenance actions return the system to the as-good-as-new state, while imperfect maintenance restores the system to a state between as-good-as-new and as-bad-as-old. In general, according to the studies conducted in this field and the reviewed research works, they can be categorized based on the following items, which are also presented in Table 1. In the production system column, the reviewed researches are categorized based on the number of units in the system, which can be single units, two units, or multiple units. In relation to maintenance strategies, most of the research conducted in this field use condition-based maintenance, which can be classified into two categories, corrective and preventive maintenance, according to the state of the system at the time of maintenance. Another thing by which past research can be categorized is maintenance delay. In some production systems, maintenance delay has been considered, and in some other researches, maintenance actions are carried out instantaneously and there is no maintenance delay time.

**Table 1:** Research summary.

Author(s)	Year	Production system	Maintenance strategy	Maintenance delay	Maintenance effect	Relationship between production rate and failure rate	Model	Solution method	Time horizon	Demand
Broek et al	2021	single-unit	CBM (Preventive and Corrective)	✓	Preventive: imperfect Corrective: perfect	✓	MDP	DP (Value iteration)	Finite	✗
Broek et al	2021	Two-unit	CBM (Preventive and Corrective)	✓	perfect	✓	MDP	DP (Value iteration policy iteration)	Infinite	✗
Koopmans and Jonge	2023	Multi-unit	CBM (Preventive and Corrective)	✗	perfect	✓	MDP	DP (Value iteration)	Infinite	✗
Mohammadipour et al	2025	single-unit	CBM (Preventive and Corrective)	✗	perfect	✓	MDP	DP (Value iteration)	Finite	✓
Keizer et al	2017	Multi-unit	CBM (Preventive and Corrective)	✓	perfect	✗	MDP	DP (Value iteration)	Infinite	✗
Broek et al	2019	single-unit	✗	✗	✗	✓	MDP	DP (Value iteration)	Finite	✗
Sun et al	2023	single-unit	Preventive	✗	✗	✓	MIP	Robust	Finite	✗
Drent et al	2023	single-unit	✗	✓	✗	✓	MDP	DP (Value iteration)	Finite	✗
Zhang et al	2024	single-unit	CBM (Preventive and Corrective)	✓	✗	✗	MDP	DP (Value iteration)	Infinite	✓
current study	2026	single-unit	CBM (Preventive and Corrective)	✓	perfect	✓	MDP	DP (Value iteration)	Finite	✓

In addition, in some researches, the relationship between failure rate and production rate has been considered and, in some researches, this relationship has been ignored. The models presented in the research in this field are often MDP based on the nature of randomness, which are solved by existing algorithms in dynamic programing and reinforcement learning such as value iteration, policy iteration, Q-learning, etc. Finally, issues can be classified according to the presence or absence of demand in the production system.

Based on a thorough review of the existing literature on condition-based maintenance and production planning, several research gaps were identified. Most prior studies have either concentrated on maintenance or production decisions in isolation, often assuming simplified conditions such as immediate maintenance actions or neglecting demand requirements. To address these limitations, this study makes the following contributions:

- Integration of condition-based maintenance and production decisions: Unlike most previous studies that focus exclusively on either maintenance or production, this research develops a unified framework that simultaneously optimizes both decisions under uncertainty.
- Consideration of customer demand in the production system: The model incorporates predetermined demand in each period, ensuring that production and maintenance decisions are aligned with real operational requirements.
- Incorporation of maintenance delays: The proposed model explicitly accounts for maintenance delays, thereby addressing a more realistic setting that is often overlooked in the existing literature.
- Formulation as a finite-horizon Markov Decision Process: The problem is modeled within the framework of a finite-horizon Markov Decision Process (MDP), enabling the derivation of optimal dynamic policies for joint production and maintenance planning.
- Development and evaluation of a dynamic policy: A dynamic Condition-Based Maintenance and Production (CBMP) policy is designed and its performance is systematically compared with conventional static policies, demonstrating superior cost-effectiveness and reliability.
- Comparative analysis under different maintenance delay scenarios: The proposed framework is applied to both zero-delay and nonzero-delay settings, providing valuable managerial insights and highlighting the robustness of the dynamic CBMP policy across different operational environments.

## 3. Problem statement

Consider a production system with an adjustable production rate. More specifically, the system has n levels of production rate and the production rate at level K’th, K={0, 1, 2,…,n}, is $\omega_{K}$ so that $\text{if }K<K^{\prime }\text{ then }\omega_{K}<\omega_{K^{\prime }}$. Level 0 is an idle state which means stoppage of the production, i.e., $\omega_{\text{0}}=\text{0},$ and level n’th means that production rate is at the maximum level.

Production rate affects the deterioration of the system, so that at a higher production rate, the expected deterioration of the system increases. From the machine deterioration point of view, it is assumed that the machine has m operational states and a non-operational state. The machine’s deterioration level will be indicated by the parameter i, which will be defined as the set {0, 1, 2,…,m}. State 0 is the as-good-as-new state, and state m is the failed state, which means the system is in failure and cannot produce items. At the K’th level of production rate the deterioration of the system is governed by a Markov chain matrix denoted as $P^{K}$ in Equation 1. The number of system deterioration modes, as mentioned, is equal to m+1, so the transition probability matrix $P^{K}$ is an (m+1)*(m+1) matrix.


$$\begin{array}{c} P^{K}=(\begin{array}{cccccc} p_{\text{0},\text{0}}^{K} & p_{\text{0},\text{1}}^{K} & p_{\text{0},\text{2}}^{K} & \cdots & p_{\text{0},m-\text{1}}^{K} & p_{\text{0},m}^{K}\\ \text{0} & p_{\text{1},\text{1}}^{K} & p_{\text{1},\text{2}}^{K} & & p_{\text{1},m-\text{1}}^{K} & p_{\text{1},m}^{K}\\ \text{0} & \text{0} & p_{\text{2},\text{2}}^{K} & & p_{\text{2},m-\text{1}}^{K} & p_{\text{2},m}^{K}\\ \vdots & & & \ddots & & \vdots \\ \text{0} & \text{0} & \text{0} & & p_{m-\text{1},m-\text{1}}^{K} & p_{m-\text{1},m}^{K}\\ \text{0} & \text{0} & \text{0} & \cdots & \text{0} & \text{1} \end{array})\text{ or }p_{i,j}^{K}=\left\{ \begin{array}{cc} \text{1} & \;if\;i=j=m\\ \text{0} & \;if\;i=m,\;j<m\\ \text{0} & \;if\;i<m,\;i>j\\ p_{i,j}^{K} & \;if\;i<m,\;i\leq j \end{array}\right. \end{array}$$ (1)

So that $p_{i,j}^{K}$is the probability of the machine transitioning from state i to j given that the production rate is at level K. If the production rate is 0, i.e., the system does not work and the production is stopped, then the machine does not deteriorate. If the machine enters deterioration level m’th, it remains there until a corrective maintenance (CM) action is conducted. Matrix $P^{K}$ is upper triangular, which means that the deterioration level of the machine cannot improve unless a maintenance action is conducted.

In each period, according to the production rate in that period t ($\omega_{K}$) and a parameter that is defined as the production constant (B), the amount of production in that period will be determined, which is presented in Equation 2. In Equation 2, B is the constant parameter of production and will be considered by a series of parameters such as equipment power, period length, working hours, etc.


$$\begin{array}{c} Z_{t}=\omega_{K}*B \end{array}$$ (2)

At the start of each decision epoch, after observing the deterioration state of the machine, the following maintenance actions can be scheduled. If the machine is in state m, the CM should be planned, otherwise, the operators can decide whether to plan a preventive maintenance (PM) action or not. There exists a deterministic maintenance delay so that if PM/CM is planned at the start of period t’th, maintenance action is conducted at the start of period t+δ while δ is the maintenance delay. Scheduling maintenance action incurs a fixed cost as A. Both PM and CM actions are assumed perfect, and they renew the machine to the as-good-as-new state. Also, it is assumed that the maintenance operations (CM/PM) require one period to be completed.

**Fig 1:**
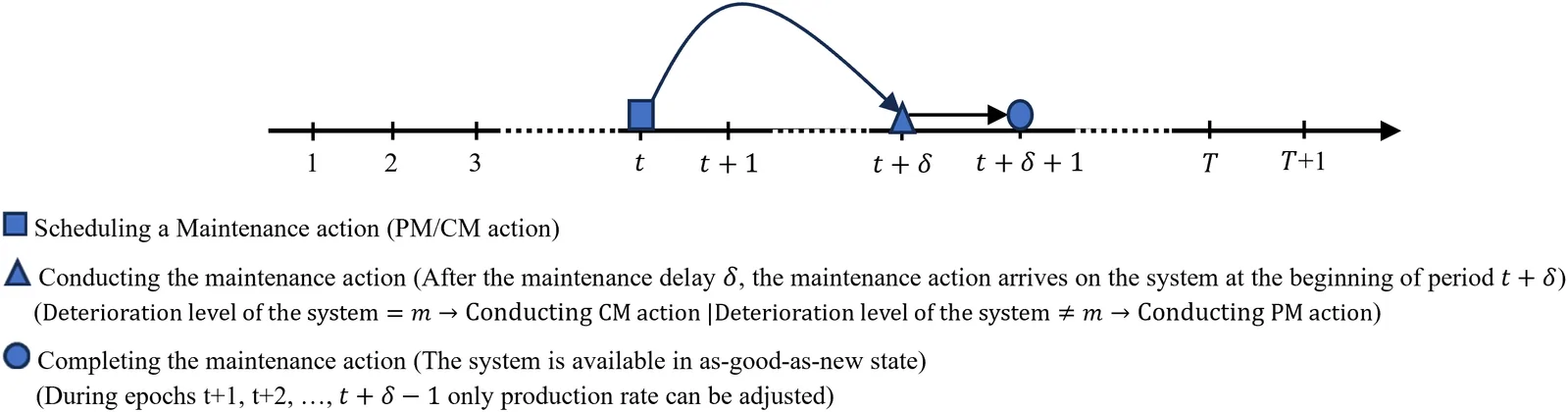
Steps to perform maintenance actions on the system.

It is important to distinguish between periodic and partial observability. In this study, the deterioration level of the machine is assumed to be fully and accurately observed at the beginning of each decision epoch. Consequently, although observations occur at discrete points in time rather than continuously, there is no uncertainty regarding the system’s true state at each decision epoch, which justifies the standard (fully observed) MDP formulation rather than a Partially Observable MDP (POMDP). In Fig 1, the steps involved in performing maintenance actions on the system are illustrated.

Since the system under consideration consists of a single deteriorating machine, at most one maintenance action (PM or CM) can ever be scheduled or awaited at any given time; consequently, contention among multiple concurrent maintenance requests for limited technician/repair capacity does not arise within the scope of the present single-unit formulation. Moreover, the maintenance delay δ explicitly incorporated in this study is intended to represent, in an aggregate manner, the time required to mobilize the resources necessary to carry out a maintenance action — including technician availability, coordination, and the procurement or arrival of spare parts — once a PM/CM action has been scheduled. In this sense, rather than ignoring resource-related constraints, the model captures their net effect on the timing of maintenance execution through the delay parameter δ, providing a tractable representation of this practical limitation without requiring an additional resource-capacity state dimension.

In addition to the planning of maintenance action, at the start of each epoch, the operator can adjust (increase or decrease) the production rate of the machine. Hence, at the start of each epoch, the following actions can be taken by the operator:

(1) If the deterioration state of the machine is m (failed state), which means the machine is in a non-operational state, the operator can:
  - CM can be planned.
  - Do nothing
(2) If the deterioration state of the machine is not m, which means the machine is in an operational state, the operator can:
  - Adjust the production rate to any level of admissible production rates from K=(0, 1, 2,…,n);
  - Planning the PM action;
  - Do nothing

The planning horizon includes T+1 decision epochs, and the prespecified demand of period t is $\varphi_{t}$. For each period, if the total production during that epoch is below the demand, two costs incur, a fixed cost associated with not reaching the demand denoted as $\pi_{\text{0}}$, and a variable cost denoted as $\pi_{\text{1}}$, which is proportional to the amount of shortage. In this problem, it is assumed that any surplus production beyond the period’s demand is sold immediately in the market at a unit bonus $\pi_{\text{2}}$., and no inventory is carried over to subsequent periods. This assumption reflects production environments in which output cannot be economically or physically stored and must be disposed of in the same period it is produced. Several real-world production systems exhibit this characteristic. For instance, in electricity generation, power produced beyond the contracted or forecasted demand is fed into the grid and sold immediately at the spot market price, since large-scale electricity storage remains costly and is not used in most conventional generation systems. Similarly, in ready-mix concrete production, output exceeding the scheduled order for a construction project must be sold to another buyer or disposed of within a short time window, as the product becomes unusable shortly after mixing. In industrial bakery production, bread manufactured beyond the day’s forecasted demand is typically sold the same day at a discounted price rather than stored for the next period, owing to its short shelf life. These examples illustrate that the assumption of immediate, no-inventory disposal of surplus production is representative of a broad class of production systems in which demand is predetermined and the product is perishable, storage-costly, or economically impractical to hold. We acknowledge that in make-to-stock environments where surplus output can be economically stored, inventory holding costs would need to be explicitly modeled; we discuss this as a limitation and a direction for future work in Section 8. According to the problem assumptions, at the start of the planning horizon, the system is available at the as-good-as-new state. Also, at the end of the planning horizon, i.e., at the beginning of period T+1, the system will undergo a mandatory maintenance action so that it returns to the as-good-as-new state. For the T+1’th epoch, there is no demand and only maintenance action is conducted. The aim of this research is to develop a Markov decision process model to minimize the operational costs of this system during the finite planning horizon. In Table 2, the notations used in the problem are presented.

**Table 2:** Nomenclature.

$\omega_{K}$	Production rate for each period, given the K’th production level.
$Z_{t}$	Production quantity in period t
$\pi_{\text{0}}$	A fixed cost associated with not reaching the demand of that period
$\pi_{\text{1}}$	The costs of shortage for a unit of demand for an epoch
$\pi_{\text{2}}$	The bonus of overproduction for a unit of production surpasses the demand of that period
A	A fixed cost for scheduling maintenance actions
$\varphi_{t}$	Demand of period t
δ	Maintenance delay
$C_{CM}$	Costs of Corrective Maintenance
$C_{PM}$	Costs of Preventive Maintenance
$p_{i,j}^{K}$	Probability of transition from state i to j given that the production rate is set at level K
$P^{K}$	Markov chain transition matrix given that the production rate is set at level K
T+1	Length of planning horizon

## 4. Derivation of the Markov decision process

In the following, the MDP model of the problem is presented. A Markov Decision Process is a mathematical framework for modeling decision-making in situations where outcomes are partly random and partly under the control of a decision-maker. MDPs are defined by four main components: states, actions, transition probabilities, and costs. The state represents the current situation or configuration of the system, while the action is a choice that affects the system’s evolution. Transition probabilities describe the likelihood of moving from one state to another given a specific action, encapsulating the stochastic nature of the process. Costs quantify the immediate expense or penalty associated with taking an action in a given state, guiding the decision-maker toward the most economical course of action. In an MDP, the objective is to develop a policy or strategy mapping states to actions that minimizes the expected cumulative cost over the planning horizon.

### 4.1. Problem state

The system deterioration level is used as the main parameter to define the problem state. Moreover, the duration until the maintenance actions arrive is also an important parameter, and in any state, the maintenance delay should be known.

On the other hand, considering that the length of the planning horizon is finite, it is necessary to have information about the current epoch in any case. Therefore, in the presented problem, the state of the problem is defined by a three-element vector: deterioration level, remaining epochs until the end of the current scheduled maintenance plan, and current epoch. Therefore, the state of the system will be represented as follows:


$$\begin{array}{c} System\;state=(i,\;\tau ,\;t) \end{array}$$

$$\begin{array}{c} i\in \left\{\text{0},\text{1},\ldots ,m\right\};\;\tau \in \left\{\text{0},\text{1},\text{2},\ldots ,\delta -\text{1},\psi \right\};\;t\in \{\text{1},\text{2},\ldots ,T+\text{1}\} \end{array}$$ (3)

Where i is the level of the system deterioration, τ is the remaining epochs until the end of the current scheduled maintenance plan, and t is the current epoch. The deterioration level of the system (i) belongs to this set, i∈{0,1,2,…,m} where state 0 means as-good-as-new state and state m means that the system is disabled and in a non-operational state.

The delay in the maintenance operation will also be represented as a set of τ∈{0,1, 2,…,δ−1, ψ}, where τ=0  indicates that the maintenance operation arrives at the system; and maintenance actions will be performed. Also, τ=ψ  means that no decision has been made to perform maintenance operations in the system. Therefore, whenever maintenance actions are planned, after a δ period, the maintenance actions will be performed on the system. It should be noted that for set τ, value δ is not included because in practice it will never occur.

On the other hand, t belongs to the set {1, 2,…,T+1}, where the state t=T+1 means the end of the planning horizon (terminal state) and the state t=1 means that the system is at the beginning of the planning horizon.

### 4.2. Problem actions

At the start of each decision epoch, according to the system state, the following actions can be taken:

(1) If the deterioration level of the machine is m (failed state), which means the machine is in a non-operational state, the operator can:
  - CM can be planned. In this case, after the arrival of maintenance actions on the system, performing corrective maintenance on the system requires one period of time, and after one period of time, the system will be available in as-good-as-new state.
  - Do nothing.
(2) If the deterioration level of the machine is not m, which means the machine is in an operational state, the operator can:
  - Adjust the production rate to any level of the admissible production rates from K={0,1,…, n}.
  - Planning the maintenance actions: In this case, after the arrival of maintenance actions on the system, the state of the machine is determined, and if it is in state m, the CM action is conducted; otherwise, the PM action is conducted. After conducting the PM/CM actions, for the next epoch, the system is in the “as-good-as-new state”. such as corrective maintenance, the actions of preventive maintenance require one period of time, and after a period, the system will be available “as-good-as-new” state.
  - Do nothing.

### 4.3. Recursive functions

In the following, the recursive equations of the MDP model are derived. Different scenarios may occur during the planning horizon, and for each scenario, different actions are available, and also the form of recursive equations is different. According to the problem assumptions, the evolution of the system during the planning horizon can be divided into 8 scenarios. In the following, for each scenario, the recursive function is derived, and suitable explanations are provided.

#### • Scenario 1.

Consider a scenario where we are at the start of period t*,* so that the number of periods remaining until the end of the planning horizon is greater than the maintenance delay time (i.e.,T−t>δ). The system deterioration level is i (i≠m) and no maintenance actions have been previously planned which means τ=ψ. For this scenario, two general actions are available (I) adjusting the production rate, and (II) planning maintenance actions. Consequently, Equation 4 recursive function is derived:


$$\begin{array}{c} V(i,\;\psi ,\;t)=Min\left\{\begin{array}{c} @c\underset{k\in K}{Min}\left\{\pi_{\text{0}}.y_{t}+\pi_{\text{1}}.max\left\{\text{0},\varphi_{t}-Z_{t}\;\right\}-\pi_{\text{2}}.max\left\{\text{0},Z_{t}-\varphi_{t}\;\right\}+\sum_{j=i}^{m}P_{ij}^{k}\;V(j,\;\psi ,\;t+\text{1})\right\}\\ ,\\ \underset{k\in K}{Min}\left\{{A+\pi }_{\text{0}}.y_{t}+\pi_{\text{1}}.max\left\{\text{0},\varphi_{t}-Z_{t}\;\right\}-\pi_{\text{2}}.max\left\{\text{0},Z_{t}-\varphi_{t}\;\right\}+\sum_{j=i}^{m}P_{ij}^{k}\;V(j,\;\delta -\text{1},\;t+\text{1})\right\}\; \end{array}\right\}\;\;\;\;\;\;\;\;\;\;\;\;\;\;\;\;\;\;\;\;\;\;\;\;\;\;\;\;\;\;\;\;\;\;\;\;\;\;\;\;\;\;\;\;\;\;\;\;\;\;\;\;\;\;\;\;\;\;\;\;\;\;\;\;\;\;\;\;\;\;\;\;\;\;\;\;\;\;\;\;\;\;\;\;\;\;\;\;\;\;\;\;\;\;\;\;\;\;\;\;\;\;\;\;\;\;\;\;\;\;\;\;\;\;\;\;\;\;\;\;\;\;\;\;\;\;\;\;\;\;\;\;\;\;\;\;\;\;\;\;\;\;\;\;\;\;\;\;\;\;\;\;\;\;\;\;\;\;\;\;\;\\ \forall i\neq m;\;T-\;t>\delta ;\;t\neq T+\text{1} \end{array}$$ (4)

$$\begin{array}{c} y_{t}=\left\{ \begin{array}{c} @l\text{1}\;if\;\varphi_{t}>Z_{t}\\ \text{0}\;otherwise \end{array}\right. \end{array}$$

#### • Scenario 2.

Scenario 2 is similar to Scenario 1, except that in this scenario, the number of periods remaining until the end of the planning horizon is less than or equal to the maintenance delay time (i.e.,T−t≤δ); therefore, in this case, it does not make sense to schedule maintenance actions. The only action is to adjust the production rate or do nothing. At the end of the period, as in scenario 1, the system deterioration state is equal to j∈{i, i+1, i+2, …, m}, and one period will be added. Consequently, the following recursive equation is derived.


$$\begin{array}{c} V(i,\;\psi ,\;t)=\underset{k\in K}{\text{min}}\left\{\pi_{\text{0}}.y_{t}+\pi_{\text{1}}.max\left\{\text{0},\varphi_{t}-Z_{t}\;\right\}-\pi_{\text{2}}.max\left\{\text{0},Z_{t}-\varphi_{t}\;\right\}+\sum_{j=i}^{m}P_{ij}^{k}\;V(j,\psi \;,\;t+\text{1})\right\}\;\forall i\neq m;\;;\;T-t\leq \delta \;;\;t\neq T+\text{1} \end{array}$$ (5)

#### • Scenario 3.

In this scenario, the system is in the operational state i’th at the beginning of period t*,* maintenance operations have been scheduled in advance, and τ periods remain until maintenance is due on the system (i.e., maintenance is scheduled at the start of period t−(δ−τ)). In this state, the only possible action to control the deterioration level of the system is to adjust the production rate. Consequently, the following recursive equation is derived:


$$\begin{array}{c} V(i,\;\tau ,\;t)=\underset{k\in K}{\text{min}}\left\{{\pi_{\text{0}}.y_{t}+\pi }_{\text{1}}.max\left\{\text{0},\varphi_{t}-Z_{t}\;\right\}-\pi_{\text{2}}.max\left\{\text{0},Z_{t}-\varphi_{t}\;\right\}+\sum_{j=i}^{m}P_{ij}^{k}\;V(j,\;\tau -\text{1},\;t+\text{1})\right\}\;\;\;\;\;\;\;\;\;\;\;\;\;\;\;\;\;\;\;\;\;\;\;\;\;\;\;\;\;\;\;\;\;\;\;\;\;\;\;\;\;\;\;\;\;\;\;\;\;\;\;\;\;\;\;\;\;\;\;\;\;\;\;\;\;\;\;\;\;\;\;\;\;\;\;\;\;\;\;\;\;\;\;\;\;\;\;\;\;\;\\ \forall \;i\neq m;\;\tau \epsilon \left\{\text{1},\;\text{2},\;\text{3},\;\ldots ,\delta -\text{1}\right\};t\neq T+\text{1} \end{array}$$ (6)

#### • Scenario 4.

In this scenario, the system is at the beginning of period t (t≠T+1), and the system has failed (i=m), the maintenance actions have already been planned, and τ periods are left until the maintenance reaches the system. In this case, because the production is stopped, the demand will not be responded and the system will have to pay a penalty, which is given in Equation 7. In this case, the only possible action is to do nothing, and the production rate is zero.


$$\begin{array}{c} V(m,\;\tau ,\;t)=\tau \pi_{\text{0}}{+\pi }_{\text{1}}\sum_{j=t}^{t+\tau }\varphi_{j}+\;V(m,\;\text{0},\;t+\tau )\;;\;\tau \epsilon \left\{\text{1},\;\text{2},\;\text{3},\;\ldots ,\delta -\text{1}\right\};\;t\neq T+\text{1}\; \end{array}$$ (7)

#### • Scenario 5.

In this scenario, the system receives pre-planned maintenance operations at the beginning of period t, i.e., the second element of the state vector is zero (τ=0). It means maintenance has been previously planned at the start of epoch t−δ. As the system is under maintenance actions, there is no production, the demand will not be responded in this period, and the system will be subject to a penalty. The decision to perform corrective or preventative maintenance will be made based on the level of system deterioration. If the system is in operational state, the preventive maintenance will be performed on the system, and if the system is failed, the corrective maintenance will be performed on the system. Both maintenance strategies are assumed to be perfect, and after performing them, after one period, the system deterioration will be changed to an as-good-as-new state. In this scenario, if the system is in as-good-as-new state (i=0), no maintenance will be performed on the system and the system will be available from the as-good-as-new state in the next period. Consequently, the following recursive equation holds:


$$\begin{array}{c} (i,\;\text{0},\;t)=\left\{ \begin{array}{cc} \;C_{cm}+\pi_{\text{0}}+\pi_{\text{1}}\varphi_{t}+V(\text{0},\psi ,t+\text{1}) & \;if\;i=m\\ C_{pm}+\pi_{\text{0}}+\pi_{\text{1}}\varphi_{t}+V(\text{0},\psi ,t+\text{1}) & \;if\;\text{0}<i<m\\ \pi_{\text{0}}+\pi_{\text{1}}\varphi_{t}+V(\text{0},\psi ,t+\text{1}) & \;if\;i=\text{0} \end{array}\right.\;\forall \;t\;\epsilon \left\{\delta +\text{1},\delta +\text{2},\delta +\text{3}\;\ldots ,T\right\} \end{array}$$ (8)

#### • Scenario 6.

The system is at the beginning of period t, and the number of periods until the end of the planning horizon is greater than the maintenance delay (T−t>δ). In this scenario, the system is in the non-operational state, and production has stopped. Maintenance operations have not been previously planned. Therefore, in this scenario, the only possible action is to schedule corrective maintenance or do nothing. In Equation 9, the recursive function related to this scenario is provided.


$$\begin{array}{c} V(m,\psi ,t)=Min\;\left\{A+\pi_{\text{0}}+\pi_{\text{1}}\varphi_{t}+V(m,\delta -\text{1},t+\text{1}),\pi_{\text{0}}+\pi_{\text{1}}\varphi_{t}+V(m,\psi ,t+\text{1})\right\}\;;if\;T-t>\delta \end{array}$$ (9)

#### • Scenario 7.

Scenario 7 is similar to Scenario 6 except that the number of periods remaining until the end of the planning horizon in this scenario is less than or equal to the maintenance delay (T−t≤δ). Therefore, in this case, the only action is “do nothing”. The system is stopped until the end of the planning horizon, and the production rate is zero. In this scenario, the system will incur a penalty for the number of periods remaining until the end of the planning horizon because the demand does not respond.


$$\begin{array}{c} V(m,\psi ,t)=\pi_{\text{0}}(T-t)+\pi_{\text{1}}\sum_{j=t}^{T}\varphi_{j}+V(m,\psi ,T+\text{1});\;if\;(T-t)\leq \delta \end{array}$$ (10)

#### • Scenario 8.

According to the assumption of the problem, the system should be restored to the as-good-as-new at the end of the planning horizon, Consequently, at the start of the T+1 epoch, if the system is in an operational state, preventive maintenance actions will be performed, and if the system is failed, the corrective maintenance action will be performed on the system. At the end of the planning horizon, if the system is in as-good-as-new state, nothing will be done on the system.


$$\begin{array}{c} V(i,\psi ,T+\text{1})=\left\{ \begin{array}{c} @lC_{CM}\;if\;i=m\\ C_{PM}\;if\;\text{0}<i<m\;\\ \text{0}\;if\;i=\text{0} \end{array}\right. \end{array}$$ (11)

As stated above, the set of states can be partitioned into 8 scenarios. Table 3 provides these scenarios along with pertinent descriptions.

**Table 3:** Scenarios with possible actions.

Scenario	Recursive function	Description	Possible Action’s
1	$\begin{array}{cc} V(i,\;\psi ,\;t)= & Min\{\underset{k\in K}{Min}\{\pi_{\text{0}}.y_{t}+\pi_{\text{1}}.max\left\{\text{0},\varphi_{t}-Z_{t}\;\right\}-\\ & \pi_{\text{2}}.max\left\{\text{0},Z_{t}-\varphi_{t}\;\right\}+\sum_{j=i}^{m}P_{ij}^{k}\;V(j,\;\psi ,\;t+\text{1})\},\\ & \underset{k\in K}{Min}\;\{\pi_{\text{0}}.y_{t}+\pi_{\text{1}}.max\left\{\text{0},\varphi_{t}-Z_{t}\;\right\}-\pi_{\text{2}}.max\left\{\text{0},Z_{t}-\varphi_{t}\;\right\}\\ & +\sum_{j=i}^{m}P_{ij}^{k}\;V(j,\;\delta -\text{1},\;t+\text{1})\}\;\} \end{array}$ ∀i≠m; T− t>δ; t≠T+1	The system is in an operational state, the maintenance actions have not previously been planned, and the number of periods remaining until the end of the planning horizon is greater than the maintenance delay.	• Planning the maintenance action • Adjusting the production rate
2	$\begin{array}{cc} V(i,\;\psi ,\;t)=\; & \underset{k\in K}{\text{min}}\{\pi_{\text{0}}.y_{t}+\pi_{\text{1}}.max\left\{\text{0},\varphi_{t}-Z_{t}\;\right\}-\pi_{\text{2}}.max\left\{\text{0},Z_{t}-\varphi_{t}\;\right\}\\ & \;\;+\sum_{j=i}^{m}P_{ij}^{k}\;V(j,\psi \;,\;t+\text{1})\}\; \end{array}$ ∀i≠m; ; T−t≤δ ; t≠T+1	The system is in an operational state, the maintenance actions have not previously been planned, and the number of periods remaining until the end of the planning horizon is less than or equal to the maintenance delay.	• Adjusting the production rate
3	$\begin{array}{cc} V(i,\;\tau ,\;t)= & \;\underset{k\in K}{\text{min}}\{{\pi_{\text{0}}.y_{t}+\pi }_{\text{1}}.max\left\{\text{0},\varphi_{t}-Z_{t}\;\right\}\\ & -\pi_{\text{2}}.max\left\{\text{0},Z_{t}-\varphi_{t}\;\right\}\\ & +\sum_{j=i}^{m}P_{ij}^{k}\;V(j,\;\tau -\text{1},\;t+\text{1})\} \end{array}\;$ ∀ i≠m; τϵ{1, 2, 3, …,δ−1};t≠T+1	The system is in an operational state and maintenance operations are scheduled in advance.	• Adjusting the production rate
4	$V(m,\;\tau ,\;t)=\tau \pi_{\text{0}}{+\pi }_{\text{1}}\sum_{j=t}^{t+\tau }\varphi_{j}+\;V(m,\;\text{0},\;t+\tau )$ ∀ τϵ{1, 2, 3, …,δ−1}; t≠T+1	The system is in the non-operational state and the maintenance operations are scheduled in advance.	• Do nothing
5	$V(i,\;\text{0},\;t)=\left\{ \begin{array}{c} C_{cm}+\pi_{\text{0}}+\pi_{\text{1}}\varphi_{t}+V(\text{0},\psi ,t+\text{1})\;if\;i=m\\ C_{pm}+\pi_{\text{0}}+\pi_{\text{1}}\varphi_{t}+V(\text{0},\psi ,t+\text{1})\;if\;\text{0}<i<m\\ \pi_{\text{0}}+\pi_{\text{1}}\varphi_{t}+V(\text{0},\psi ,t+\text{1})\;if\;i=\text{0} \end{array}\right.\;$ ∀ t ϵ{δ+1,δ+2,δ+3 …,T}	Start of conducting the maintenance operations (PM/CM) on the system, i.e., τ=0. If i=0, nothing will be done on the system.	• Performing maintenance operations on the system • Do nothing
6	$\begin{array}{cc} V(m,\psi ,t)=\; & Min\;\{A+\pi_{\text{0}}+\pi_{\text{1}}\varphi_{t}+V(m,\delta -\text{1},t\\ & +\text{1}),\pi_{\text{0}}+\pi_{\text{1}}\varphi_{t}+V(m,\psi ,t+\text{1})\}\;; \end{array}$ if T−t>δ	The system is in the non-operational state, the maintenance actions are not planned in advance, and the number of periods remaining until the end of the planning horizon is greater than the maintenance delay.	• Planning the corrective maintenance • Do nothing
7	$V(m,\psi ,t)=\pi_{\text{0}}(T-t)+\pi_{\text{1}}\sum_{j=t}^{T}\varphi_{j}+V(m,\psi ,T+\text{1});$ if (T−t)≤δ	The system is in the non-operational state, the maintenance actions are not planned in advance, and the number of periods remaining until the end of the planning horizon is less than or equal the maintenance delay.	• Do nothing
8	$V(i,\psi ,T+\text{1})=\left\{ \begin{array}{c} C_{CM}\;if\;i=m\\ C_{PM}\;if\;i\text{≠}m\\ \text{0}\;if\;i=\text{0} \end{array}\right.\;$	End of the planning horizon (terminal state)	• Performing maintenance operations on the system • Do nothing

The MDP formulation presented above addresses a single-component production system. Extending this formulation to a multi-unit system would require several substantive changes to the model. First, the system state would need to jointly track the deterioration level of every unit, i.e., $i=(i_{\text{1}},i_{\text{2}},\ldots ,i_{N})$ for N units, together with the maintenance-delay status of each unit; this causes the state space to grow multiplicatively with N, giving rise to the well-known curse of dimensionality. Second, dependencies among units would need to be explicitly modeled, including economic dependence (cost savings from performing maintenance on multiple units jointly, due to a shared fixed cost), stochastic dependence (the deterioration or failure of one unit affecting the failure behavior of others), and structural dependence (maintenance on one unit requiring other units to be stopped). Third, the action space would need to be extended: instead of an independent PM/CM decision for each unit, the decision-maker would need to determine which subset of units to maintain jointly (group or opportunistic maintenance), and production-rate decisions would need to account for interactions across units, such as load-sharing when one unit’s rate is reduced or the unit fails and the remaining units must compensate to meet demand. Finally, unlike the single-unit case considered in this study, where at most one maintenance action can be scheduled at any time, a multi-unit system may require multiple units to be maintained concurrently, in which case limited maintenance-resource (technician) capacity would become a directly relevant constraint. Given the resulting growth in state-space size, exact solution via value iteration would likely become computationally intractable for realistic multi-unit instances, motivating the use of approximate dynamic programming or reinforcement-learning-based solution methods. We identify this multi-unit extension as a valuable direction for future research.

## 5. Numerical example

We have a single-component manufacturing system whose failure rate depends on the production rate. In this system, it is assumed that whenever maintenance actions are planned, they will reach the system after a certain maintenance delay, which is considered to be 3. It means δ=3 and τ∈{0, 1, 2,ψ}. To mitigate the system degradation, a corrective or preventive maintenance can be performed. The cost of corrective maintenance is equal to $C_{CM}=\text{2}\text{0}\text{0}\text{0}$ and the cost of preventive maintenance is equal to $C_{PM}=\text{2}\text{0}\text{0}$. The fixed cost of maintenance is set at 50 (A=50).

In this problem, it is assumed that the planning horizon includes 10 periods (T=10), and during each period, there is a predetermined demand. In each period, if the demand is not responded, the system is subject to a general penalty of $\pi_{\text{0}}=\text{1}\text{0}$ monetary units and for each unit of shortage, a penalty equal to $\pi_{\text{1}}=\text{0}.\text{5}$. On the other hand, if the demand is responded, $\pi_{\text{2}}=\text{1}$ monetary unit for each unit of demand. The value of B is taken to be 1000.

It is assumed that the production system can have 5 levels of production (K=0, 1, 2, 3, 4), where level 4 means that the system works at the maximum production rate and level 0 means that the system is stopped. In other words, this system can have a production rate equals to 0, 25%, 50%, 75%, or 100% of its maximum production capacity. Also, this system has 4 failure levels (m=0, 1, 2, 3), where level 0 means that the system is in as-good-as-new state and level 3 means that the system is failed and stopped. To obtain the optimal policy and value in each case, the value iteration algorithm is used, whose pseudo-code is shown in Table 4.

**Table 4:** The algorithm of value iteration taken from [33].

Initialize array V arbitrarily (e.g., V (s)=0 for all s ∈ S +) Repeat Δ ← 0 For each s∈S: v ← V (s) $\;V\;(s)\leftarrow {max}_{a\;}\sum_{s^{\prime },r}p\left(s^{\prime },r|s,a\right)[r+\gamma V\left(s^{\prime }\right)]$ Δ ← max(Δ, |v − V (s)|) until Δ < θ (a small positive number) Output a deterministic policy, π, such that $\pi (s)\;=\;arg{max}_{a\;}\sum_{s^{\prime },r}p\left(s^{\prime },r|s,a\right)[r+\gamma V\left(s^{\prime }\right)]$

The matrices that govern the system deterioration under each production rate are provided in the following:


$$\begin{array}{cc} & P^{\text{0}}=[\begin{array}{cccc} \text{1} & \text{0} & \text{0} & \text{0}\\ \text{0} & \text{1} & \text{0} & \text{0}\\ \text{0} & \text{0} & \text{1} & \text{0}\\ \text{0} & \text{0} & \text{0} & \text{1} \end{array}]\;P^{\text{1}}=[\begin{array}{cccc} \text{0}.\text{5} & \text{0}.\text{3} & \text{0}.\text{1}\text{5} & \text{0}.\text{0}\text{5}\\ \text{0} & \text{0}.\text{5} & \text{0}.\text{3} & \text{0}.\text{2}\\ \text{0} & \text{0} & \text{0}.\text{5} & \text{0}.\text{5}\\ \text{0} & \text{0} & \text{0} & \text{1} \end{array}]\;P^{\text{2}}=\;[\begin{array}{cccc} \text{0}.\text{3} & \text{0}.\text{4} & \text{0}.\text{2} & \text{0}.\text{1}\\ \text{0} & \text{0}.\text{3} & \text{0}.\text{4} & \text{0}.\text{3}\\ \text{0} & \text{0} & \text{0}.\text{3} & \text{0}.\text{7}\\ \text{0} & \text{0} & \text{0} & \text{1} \end{array}]\;P^{\text{3}}=[\begin{array}{cccc} \text{0}.\text{1}\text{5} & \text{0}.\text{4}\text{5} & \text{0}.\text{2}\text{5} & \text{0}.\text{1}\text{5}\\ \text{0} & \text{0}.\text{1}\text{5} & \text{0}.\text{5} & \text{0}.\text{3}\text{5}\\ \text{0} & \text{0} & \text{0}.\text{1}\text{5} & \text{0}.\text{8}\text{5}\\ \text{0} & \text{0} & \text{0} & \text{1} \end{array}]\\ & {\;P}^{\text{4}}=[\begin{array}{cccc} \text{0}.\text{0}\text{5} & \text{0}.\text{5} & \text{0}.\text{3} & \text{0}.\text{1}\text{5}\\ \text{0} & \text{0}.\text{0}\text{5} & \text{0}.\text{5}\text{5} & \text{0}.\text{4}\\ \text{0} & \text{0} & \text{0}.\text{0}\text{5} & \text{0}.\text{9}\text{5}\\ \text{0} & \text{0} & \text{0} & \text{1} \end{array}] \end{array}$$

It is assumed that the demand in each period is predetermined as presented in Table 5.

**Table 5:** Demand of each period.

t	1	2	3	4	5	6	7	8	9	10
$\varphi_{t}$	500	700	100	1400	250	200	500	180	850	1500

Table 6 lists the possible actions along with their descriptions.

**Table 6:** Different available actions of the example.

Action	0	1	2	3	4
**Description**	Adjust the production rate at $\omega_{\text{0}}=\text{0}$ (Do not schedule maintenance actions)	Adjust the production rate at $\omega_{\text{0}}=\text{0}.\text{2}\text{5}$ (Do not schedule maintenance actions)	Adjust the production rate at $\omega_{\text{0}}=\text{0}.\text{5}$ (Do not schedule maintenance actions)	Adjust the production rate at $\omega_{\text{0}}=\text{0}.\text{7}\text{5}$ (Do not schedule maintenance actions)	Adjust the production rate at $\omega_{\text{0}}=\text{1}$ (Do not schedule maintenance actions)
**Action**	5	6	7	8	9
**Description**	Adjust the production rate at $\omega_{\text{0}}=\text{0}$ and schedule maintenance action	Adjust the production rate at $\omega_{\text{0}}=\text{0}.\text{2}\text{5}$ and schedule maintenance action	Adjust the production rate at $\omega_{\text{0}}=\text{0}.\text{5}$ and schedule maintenance action	Adjust the production rate at $\omega_{\text{0}}=\text{0}.\text{7}\text{5}$ and schedule maintenance action	Adjust the production rate at $\omega_{\text{0}}=\text{1}$ and schedule maintenance action
**Action**	*PM*		*CM*		
**Description**	Performing preventive maintenance operations on the system		Performing corrective maintenance operations on the system		

As δ=3, and conducting maintenance actions, i.e., PM/CM, takes one period of time, planning maintenance actions for t≥7 is not justifiable. In other words, for a specific period as *t,* and given that τ=ψ, scheduling PM/CM actions is an admissible action if t<7. Moreover, according to the problem assumptions, for a specific period, if τ≠ψ, then scheduling maintenance actions is not an admissible action. Thus, scheduling maintenance action is an available action for a period if t<7 and τ=ψ.

For a period with τ=1 and 2 , only the production rate can be adjusted. It means that for these states 5 actions are admissible. Additionally, for a period with τ=0, given the system degradation, PM, CM or “Do nothing” should be selected. The last period T+1=11, which is the end of the planning horizon, PM, CM or “Do nothing” is always conducted and the system is restores to the as-good-as-new state. To summarize the admissible actions for each state, Table 7 is provided.

**Table 7:** Admissible actions for each state in the numerical example.

Scenario	State	Admissible Actions	Total number of admissible actions
1	• i≠3 • t<7 • τ=ψ	Adjusting the production rate and whether or not to schedule maintenance actions	10 actions
2	• i≠3 • t=7, 8, 9, 10 • τ=ψ	Adjusting the production rate	5 actions
3	• i≠3 • τ=1 or 2 • t≠11	Adjusting the production rate	5 actions
4	• i=3 • τ=1 or 2 • t≠11	‘Do nothing’	1 action
5	• i=0, 1, 2, 3 • τ=0 • t=4, 5,…,10	Conduction PM, CM or ‘Do nothing’	3 actions
6	• i=3 • τ=ψ • t<7	Planning CM action or ‘Do nothing’	2 actions
7	• i=3 • τ=ψ • t=7, 8, 9, 10	‘Do nothing’	1 action
8	• t=11	Conduction PM, CM or ‘Do nothing’	3 actions

The results of the Value Iteration algorithm are reported in Tables 8–18. In these tables, the horizontal axis represents the degradation level of the system (i), and the vertical axis represents the maintenance delay (τ). In the provided tables, whenever ‘-’ is written in a cell, it means that, according to the problem assumptions, that state will not occur. For example, in Table 9, the state (1,0, 2) will never occur because the maintenance delay is 3 and if maintenance is already scheduled, it will not reach the system at t=2. In Table 8, because the system is at the beginning of the planning horizon and starts in an as-good-as-new state, the only possible state is when i=0 and τ=ψ*.*

**Table 8:** Optimal policy for each state (i, τ,1).

τ	i			
	0	1	2	3
0	–	–	–	–
1	–	–	–	–
2	–	–	–	–
ψ	4	–	–	–

**Table 9:** Optimal policy for each state (i, τ,2).

τ	i			
	0	1	2	3
0	–	–	–	–
1	–	–	–	–
2	4	3	2	0
ψ	4	4	7	5

**Table 10:** Optimal policy for each state (i, τ,3).

τ	i			
	0	1	2	3
0	–	–	–	–
1	2	2	1	0
2	2	2	1	0
ψ	4	4	7	5

**Table 11:** Optimal policy for each state (i, τ,4).

τ	i			
	0	1	2	3
0	Do nothing	PM	PM	CM
1	4	4	3	0
2	4	4	3	0
ψ	4	4	7	5

**Table 12:** Optimal policy for each state (i, τ,5).

τ	i			
	0	1	2	3
0	Do nothing	PM	PM	CM
1	3	2	1	0
2	3	2	1	0
ψ	4	3	7	5

**Table 13:** Optimal policy for each state (i, τ,6).

τ	i			
	0	1	2	3
0	Do nothing	PM	PM	CM
1	3	2	1	0
2	3	2	1	0
ψ	4	9	7	5

**Table 14:** Optimal policy for each state (i, τ,7).

τ	i			
	0	1	2	3
0	Do nothing	PM	PM	CM
1	2	2	1	0
2	3	2	1	0
ψ	4	2	1	0

**Table 15:** Optimal policy for each state (i, τ,8).

τ	i			
	0	1	2	3
0	Do nothing	PM	PM	CM
1	2	1	1	0
2	–	–	–	–
ψ	3	2	1	0

**Table 16:** Optimal policy for each state (i, τ,9).

τ	i			
	0	1	2	3
0	Do nothing	PM	PM	CM
1	–	–	–	–
2	–	–	–	–
ψ	4	3	2	0

**Table 17:** Optimal policy for each state (i, τ,10).

τ	i			
	0	1	2	3
0	–	–	–	–
1	–	–	–	–
2	–	–	–	–
ψ	4	4	4	0

**Table 18:** Optimal policy in terminal state.

τ	i			
	0	1	2	3
0	–	–	–	–
1	–	–	–	–
2	–	–	–	–
ψ	Do nothing	PM	PM	CM

In Tables 19–29 the optimal value of each state is presented. It should be noted that the numbers written in these tables represent the expected cost of each mode. For example, the optimal value of state (0, ψ, 9) shown in Table 27, is equal to 212 monetary units. It should be recalled that the state-value function of (0,ψ,1) is the expected optimal costs of the system during the planning horizon which is 1633.

**Table 19:** Optimal value for each state (i, τ,1).

τ	i			
	0	1	2	3
0	–	–	–	–
1	–	–	–	–
2	–	–	–	–
ψ	1633	–	–	–

**Table 20:** Optimal value for each state (i, τ,2).

τ	i			
	0	1	2	3
0	–	–	–	–
1	–	–	–	–
2	1584	1598	1611	1624.7
ψ	1574.1	1580	1593	1601.5

**Table 21:** Optimal value for each state (i, τ,3).

τ	i			
	0	1	2	3
0	–	–	–	–
1	1488	1539	1593	1619
2	1470.3	1528.4	1593	1619
ψ	1432	1512.6	1557.1	1597

**Table 22:** Optimal value for each state (i, τ,4).

τ	i			
	0	1	2	3
0	1305.3	1324.2	1324	1435.5
1	1275	1296.3	1309	1347
2	1235.2	1285	1289	1341.7
ψ	1212.8	1264.4	1272.5	1320

**Table 23:** Optimal value for each state (i, τ,5).

τ	i			
	0	1	2	3
0	1190.3	1246.5	1252.7	1361.7
1	1157.1	1214.2	1217.2	1237.2
2	1123	1193.9	1199	1222.7
ψ	1098	1140	1162	1203

**Table 24:** Optimal value for each state (i, τ,6).

τ	i			
	0	1	2	3
0	893.1	886.2	914.2	1020.4
1	862.4	867	869	893.1
2	850.8	867	869	892.3
ψ	841.5	853.6	861	886

**Table 25:** Optimal value for each state (i, τ,7).

τ	i			
	0	1	2	3
0	660.4	649	660.1	844
1	637	627	634	731
2	625	627	634	715.8
ψ	617.5	621	632	714.3

**Table 26:** Optimal value for each state (i, τ,8).

τ	i			
	0	1	2	3
0	502	523	539.6	637.4
1	483	493.5	497.5	527.9
2	–	–	–	–
ψ	470.3	477	488.1	514

**Table 27:** Optimal value for each state (i, τ,9).

τ	i			
	0	1	2	3
0	230.5	254.2	259.3	522.4
1	**–**	**–**	**–**	**–**
2	**–**	**–**	**–**	**–**
ψ	212	229	231.8	292

**Table 28:** Optimal value for each state (i, τ,10).

τ	i			
	0	1	2	3
0	**–**	**–**	**–**	**–**
1	**–**	**–**	**–**	**–**
2	**–**	**–**	**–**	**–**
ψ	80	80	80	273

**Table 29:** Optimal value in terminal state.

τ	i			
	0	1	2	3
0	**–**	**–**	**–**	**–**
1	**–**	**–**	**–**	**–**
2	**–**	**–**	**–**	**–**
ψ	0	0	0	0

Next, the computational complexity of the problem is examined. The size of the state space is |S|= O((m+1)(δ+1)(T+1)). Where m+1 is the number of deterioration levels, δ+1 is the number of maintenance-delay states, and T+1 is the number of decision epochs. For each state, the optimal action is selected among at most 2(n+1) admissible actions: this bound reflects the two independent decisions available in the most general scenario (Scenario 1) — selecting one of n+1 production-rate levels, and independently deciding whether or not to schedule a maintenance action — which combine multiplicatively to give 2(n+1) candidate actions. This is consistent with the numerical example of Section 5, where n=4 and the model indeed admits 2(4+1)=10 actions in Scenario 1 (Table 6), matching the count reported in Table 7; other scenarios involve fewer admissible actions (e.g., only n+1 once maintenance is no longer schedulable, or a single “do nothing” action), so 2(n+1) represents a conservative upper bound across all scenarios.

Evaluating each candidate action requires computing a summation over up to m+1 terms. Combining these elements, the overall time complexity of solving the model is: $O({\left(m+\text{1}\right)}^{\text{2}}(\delta +\text{1})(T+\text{1})(n+\text{1}))$.

The quadratic dependence on (m+1) arises from two distinct, nested sources: first, the deterioration level i  itself takes m+1 possible values, contributing a factor of (m+1) to the size of the state space; second, for each such state, evaluating a candidate action requires summing over up to m+1 possible next-period deterioration levels j. Since this inner summation is repeated for every value of i, the two factors of (m+1) multiply rather than add — analogous to the cost of multiplying an (m+1)×(m+1) transition matrix by a value vector.

Table 30 summarizes the components of this complexity analysis, together with their corresponding values for the numerical example presented in Section 5 (m = 3, δ = 3, T = 10, n = 4), illustrating that the actual problem instance solved in this study involves a modest state space (176 states) and at most 10 admissible actions per state.

**Table 30:** Computational complexity components of the proposed value iteration (backward induction) procedure.

Component	Notation	Size (general)	Value for numerical example (Sec. 5)
Deterioration levels	m+1	—	4
Maintenance-delay states	δ+1	—	4
Planning-horizon epochs	T+1	—	11
Production/action levels	n+1	—	5
**State space size**	|S|	O((m+1)(δ+1)(T+1))	4×4×11=176
**Action space size (max)**	${\left|A\right|}_{max}$	O(2(n+1))	2×5=10
Transition summation per action	—	O(m+1)	4
**Time complexity (overall)**	—	$O((m+\text{1}{)}^{\text{2}}(\delta +\text{1})(T+\text{1})(n+\text{1}))$	${\text{4}}^{\text{2}}\times \text{4}\times \text{1}\text{1}\times \text{5}=\text{3},\text{5}\text{2}\text{0}$
**Memory complexity**	—	O((m+1)(δ+1))	4×4=16

We note that this favorable complexity result is specific to the single-unit formulation considered in this study. In multi-unit extensions of the model, the state space would grow multiplicatively with the number of units, giving rise to the well-known curse of dimensionality; addressing this challenge would likely require approximate dynamic programming or reinforcement-learning-based solution methods rather than exact value iteration, and constitutes a promising direction for future research.

## 6. Application

In [31], the application of joint decisions of production planning and condition-based maintenance to control the production rate of an extruder system is investigated. An extruder uses a screw to transport molten plastic raw material through a barrel to produce bottle caps. Due to the wear of the screw, the molten material may leak into the barrel and then a thermal resistor is activated for cooling. Hence, the average daily usage frequency of the thermal resistor is used as a signal to monitor the system status. Deterioration data of an extruder in an Italian factory is collected from the date of inspection in July 2017 to July 2019. During the study period, the screw rotation speed (normalized from 0 to 1) and the degradation rate (thermal resistor usage) are recorded every month. Therefore, there are 24 observations in total. In the example presented, the failure threshold is considered to be 4 and it is assumed that the screw can rotate at three levels (K=3) or in other words, its production rate is equal to 0, 0.5 and 1. Therefore, the transition probability matrix is also presented as follows, considering the relationship between the screw rotation speed and the failure rate presented in the source [31].


$$\begin{array}{c} P^{\text{0}}=[\begin{array}{ccccc} \text{1} & \text{0} & \text{0} & \text{0} & \text{0}\\ \text{0} & \text{1} & \text{0} & \text{0} & \text{0}\\ \text{0} & \text{0} & \text{1} & \text{0} & \text{0}\\ \text{0} & \text{0} & \text{0} & \text{1} & \text{0}\\ \text{0} & \text{0} & \text{0} & \text{0} & \text{1} \end{array}]\;P^{\text{1}}=[\begin{array}{ccccc} \text{0}.\text{4} & \text{0}.\text{3} & \text{0}.\text{2} & \text{0}.\text{0}\text{5} & \text{0}\\ \text{0} & \text{0}.\text{4} & \text{0}.\text{3} & \text{0}.\text{2} & \text{0}.\text{1}\\ \text{0} & \text{0} & \text{0}.\text{4} & \text{0}.\text{4} & \text{0}.\text{2}\\ \text{0} & \text{0} & \text{0} & \text{0}.\text{7} & \text{0}.\text{3}\\ \text{0} & \text{0} & \text{0} & \text{0} & \text{1} \end{array}]\;P^{\text{2}}=[\begin{array}{ccccc} \text{0}.\text{1} & \text{0}.\text{3} & \text{0}.\text{4} & \text{0}.\text{1} & \text{0}.\text{1}\\ \text{0} & \text{0}.\text{1} & \text{0}.\text{4} & \text{0}.\text{3} & \text{0}.\text{2}\\ \text{0} & \text{0} & \text{0}.\text{1} & \text{0}.\text{6} & \text{0}.\text{3}\\ \text{0} & \text{0} & \text{0} & \text{0}.\text{2} & \text{0}.\text{8}\\ \text{0} & \text{0} & \text{0} & \text{0} & \text{1} \end{array}] \end{array}$$

Important parameters of the problem such as the demand in each period, the considered system costs, and the maintenance delay are presented in Table 31.

**Table 31:** The parameters used in the problem.

t	1	2	3	4	5	6
$\varphi_{t}$	850	115	1500	1400	250	700
*B*	$C_{CM}$	$C_{PM}$	δ	$\pi_{\text{0}}$	$\pi_{\text{1}}$	$\pi_{\text{2}}$
1000	1000	500	2	2	0.1	1

Table 32 lists possible actions related to the given application example along with a description of each action.

**Table 32:** Different available actions of the application example.

Action	0	1	2
**Description**	Adjust the production rate at$\omega_{\text{0}}=\text{0}$ (Do not schedule maintenance actions).	Adjust the production rate at $\omega_{\text{0}}=\text{0}.\text{5}$ (Do not schedule maintenance actions)	Adjust the production rate at$\omega_{\text{0}}=\text{1}$ (Do not schedule maintenance actions)
**Action**	3	4	5
**Description**	Adjust the production rate at $\omega_{\text{0}}=\text{0}$ and schedule maintenance action	Adjust the production rate at $\omega_{\text{0}}=\text{0}.\text{5}$ and schedule maintenance action	Adjust the production rate at $\omega_{\text{0}}=\text{1}$ and schedule maintenance action
**Action**	*PM*		*CM*
**Description**	Performing preventive maintenance operations on the system		Performing corrective maintenance operations on the system

The presented problem is solved using the value iteration algorithm and the optimal policies for different states are obtained. The optimal policies are shown in Tables 33–39.

**Table 33:** Optimal policy for each state (i, τ,1).

τ	i				
	0	1	2	3	4
0	–	–	–	–	–
1	–	–	–	–	–
ψ	2	–	–	–	–

**Table 34:** Optimal policy for each state (i, τ,2).

τ	i				
	0	1	2	3	4
0	–	–	–	–	–
1	2	1	1	1	0
ψ	2	1	1	4	3

**Table 35:** Optimal policy for each state (i, τ,3).

τ	i				
	0	1	2	3	4
0	Do nothing	PM	PM	PM	CM
1	2	2	2	2	0
ψ	2	2	5	5	3

**Table 36:** Optimal policy for each state (i, τ,4).

τ	i				
	0	1	2	3	4
0	Do nothing	PM	PM	PM	CM
1	2	2	2	2	0
ψ	2	2	2	2	0

**Table 37:** Optimal policy for each state (i, τ,5).

τ	i				
	0	1	2	3	4
0	Do nothing	PM	PM	PM	CM
1	–	–	–	–	–
ψ	2	1	1	1	0

**Table 38:** Optimal policy for each state (i, τ,6).

τ	i				
	0	1	2	3	4
0	–	–	–	–	–
1	–	–	–	–	–
ψ	2	2	1	1	0

**Table 39:** Optimal policy in terminal state.

τ	i				
	0	1	2	3	4
0	–	–	–	–	–
1	–	–	–	–	–
ψ	Do nothing	PM	PM	PM	CM

## 7. Sensitivity analysis

In this section, we will compare our presented CBMP dynamic policy with some static policies. To this end, six static policies will be considered for production and maintenance. Expected costs during the planning horizon, which is indeed the state-value function of the initial state of (0,ψ,1), is used as a criterion for this comparison. It is worth recalling that state (0,ψ,1)  means that we are at the start of the planning horizon, the system degradation is 0 (as-good-as-new), and no maintenance actions are previously planned.

The static policies considered are as follows:

- Policy ‘a’: Production at maximum capacity with PM/CM maintenance performed only at the 5^th^ epoch.
- Policy ‘b’: Run-to-failure maintenance with production at maximum capacity throughout the planning horizon.
- Policy ‘c’: Run-to-failure maintenance with production at 50% or 75% capacity throughout the planning horizon.
- Policy ‘d’: PM/CM actions at periods 4 and 7, with production at 75% capacity.
- Policy ‘e’: Considering production rates of 0 and 1. In this policy, the production rate is selected at its minimum and maximum levels. That is, the system is either off or produces at the maximum rate. This policy assumes that the decision to perform maintenance operations will be made dynamically.
- Policy ‘f’: Considering three production rates of 0, 0.5 and 1. This policy assumes that the decision to perform maintenance operations will be made dynamically.

To obtain the optimal value of the initial state, the value iteration algorithm has been used, in which, according to the conditions defined for each fixed policy, the code has been written and the initial state value extracted. In Table 40, the expected costs of the static policies a-f and the CBMP dynamic policy for the example of Section 5 are obtained and presented.

**Table 40:** The operational costs of the system during the planning horizon under dynamic CBMP policy and 6 static policies.

Policy	a	b	c	d	e	f	CBMP
Optimal value	2234.7	2486.1	2317	1948.2	2142	1803.2	1633

To quantify the magnitude of this improvement, the dynamic CBMP policy reduces the expected operational cost by approximately 9.4% relative to the best-performing static policy (Policy f) and by 24.2% relative to the average of the six static policies, in the case with maintenance delay (Table 40). Comparable reductions of 9.6% and 24.1%, respectively, are observed in the zero-delay case (Table 43). These reductions represent a practically meaningful improvement in operational cost, particularly considering that Policy f itself already incorporates a degree of dynamic maintenance decision-making; the improvement relative to purely static policies (e.g., Policy b) is substantially larger, reaching 34.3% and 37.4% in the delay and zero-delay cases, respectively. Furthermore, as shown in Fig 3, this cost advantage becomes more pronounced as the corrective maintenance cost increases, indicating that the practical value of the dynamic policy is greatest in higher-cost operational settings.

**Fig 2:**
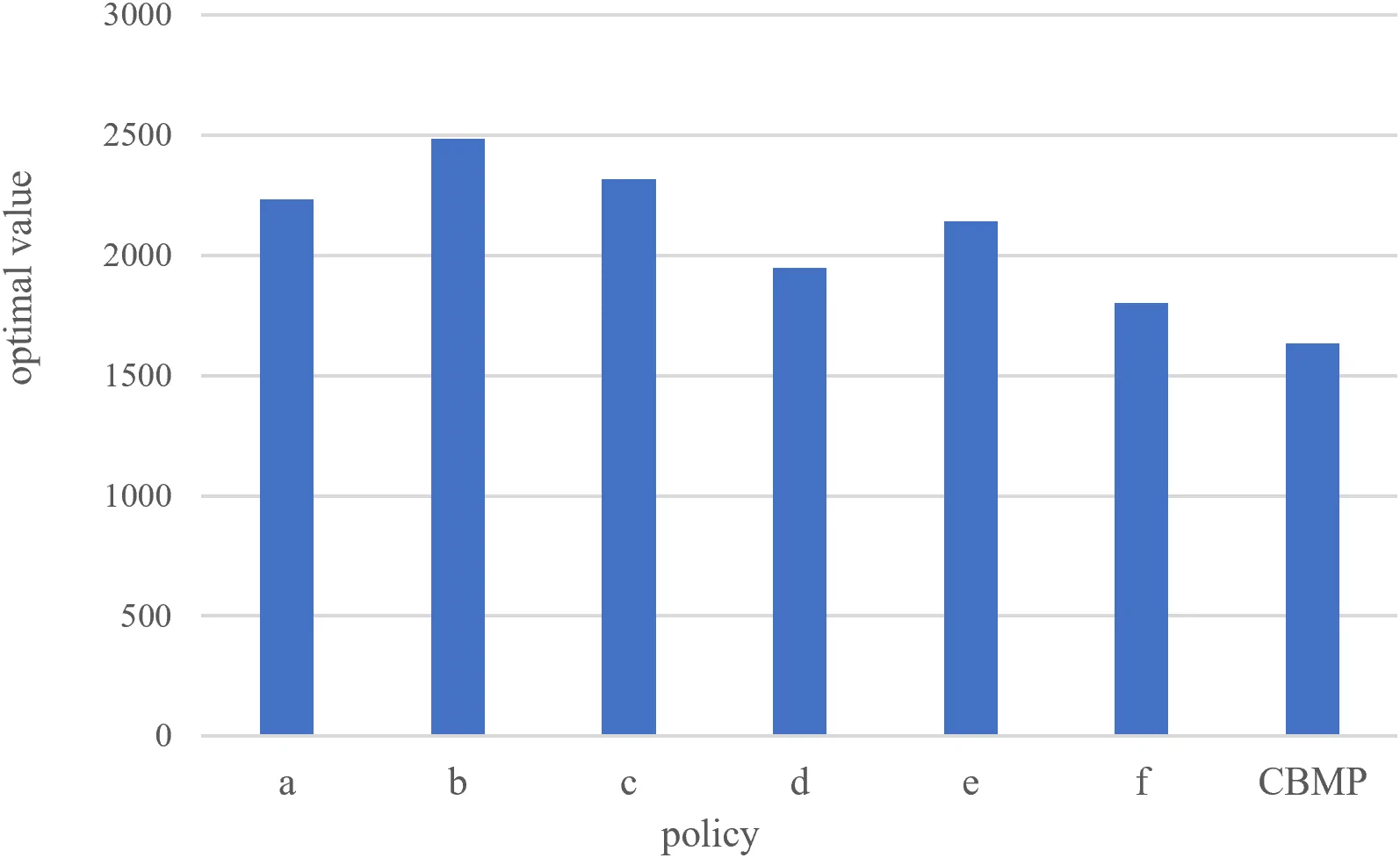
The optimal value for static policies and CBMP policy.

Fig 2 also shows these expected total costs for different policies. According to this diagram, the expected total costs during the planning horizon under the proposed CBMP dynamic policy is less than the other static policies. The reason for this is that in the dynamic CBMP policy, in each state, decisions regarding maintenance operations or adjustment of the production rate are made according to the system status and the demand that exists. Therefore, in this case, it is expected that the optimal value, which is in the form of cost, is at the lowest level compared to other constant policies.

Dynamic policy (CBMP), in which maintenance and production rate adjustment decisions are made based on system conditions, is recognized as an advanced approach in managing production systems. In this policy, unlike static policies, decisions are made based on real-time data and information on the system status, demand, and other related factors. A dynamic policy can adapt to changes in demand, system status (such as inventory levels, breakdowns, etc.), and other environmental factors. This leads to improved efficiency and reduced costs. Using real-time data, a dynamic policy can make optimal decisions regarding maintenance scheduling, production rate adjustment, and resource allocation. By preventing overproduction or shortages, and by performing timely maintenance, a dynamic policy can reduce overall system costs. By performing regular maintenance, a dynamic policy can increase system reliability and prevent sudden breakdowns. By responding to demand changes and providing quality products, a dynamic policy can improve customer satisfaction.

**Fig 3:**
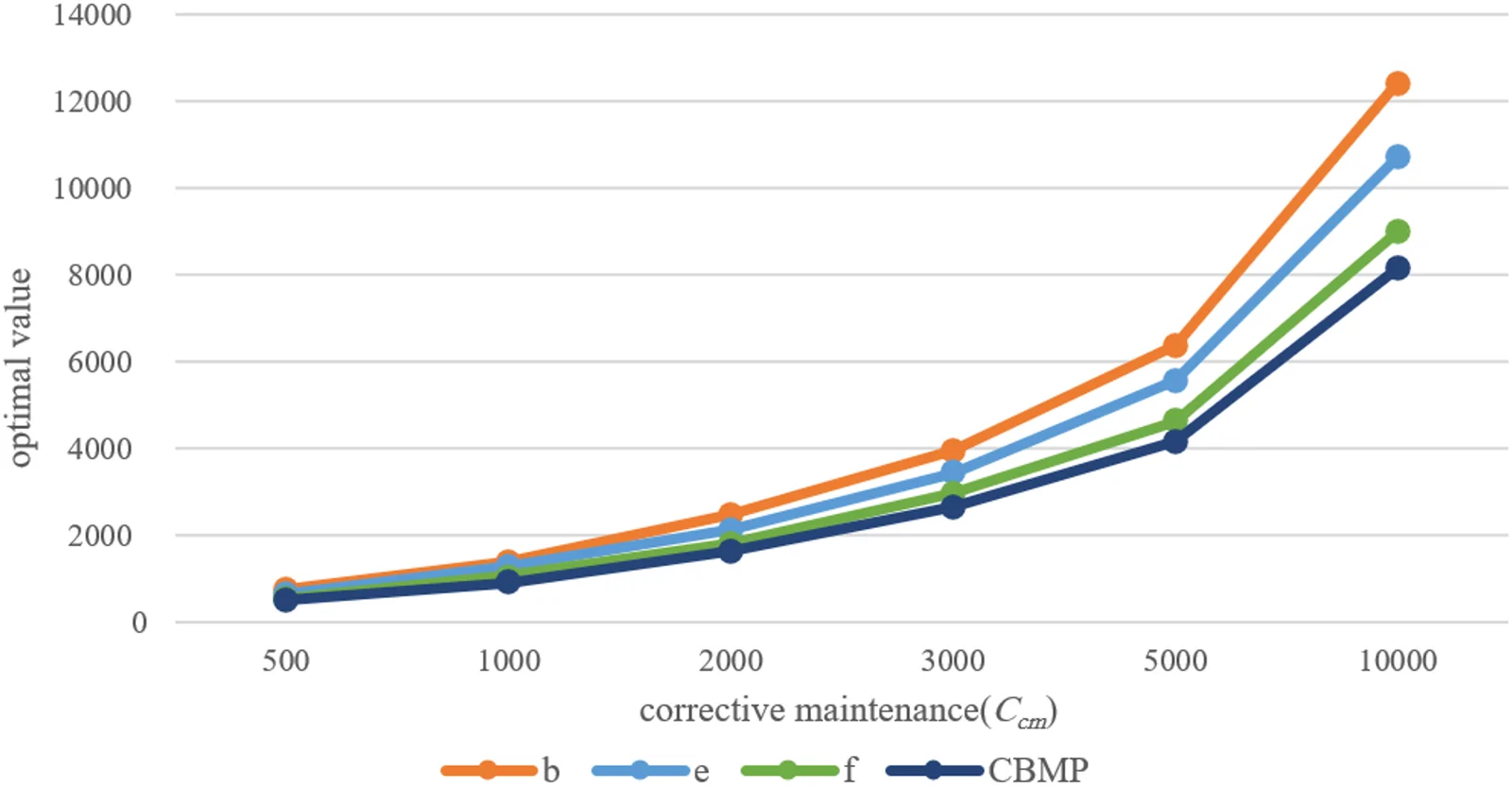
The effect of corrective maintenance cost on the optimal value under different policies (static and dynamic).

**Fig 4:**
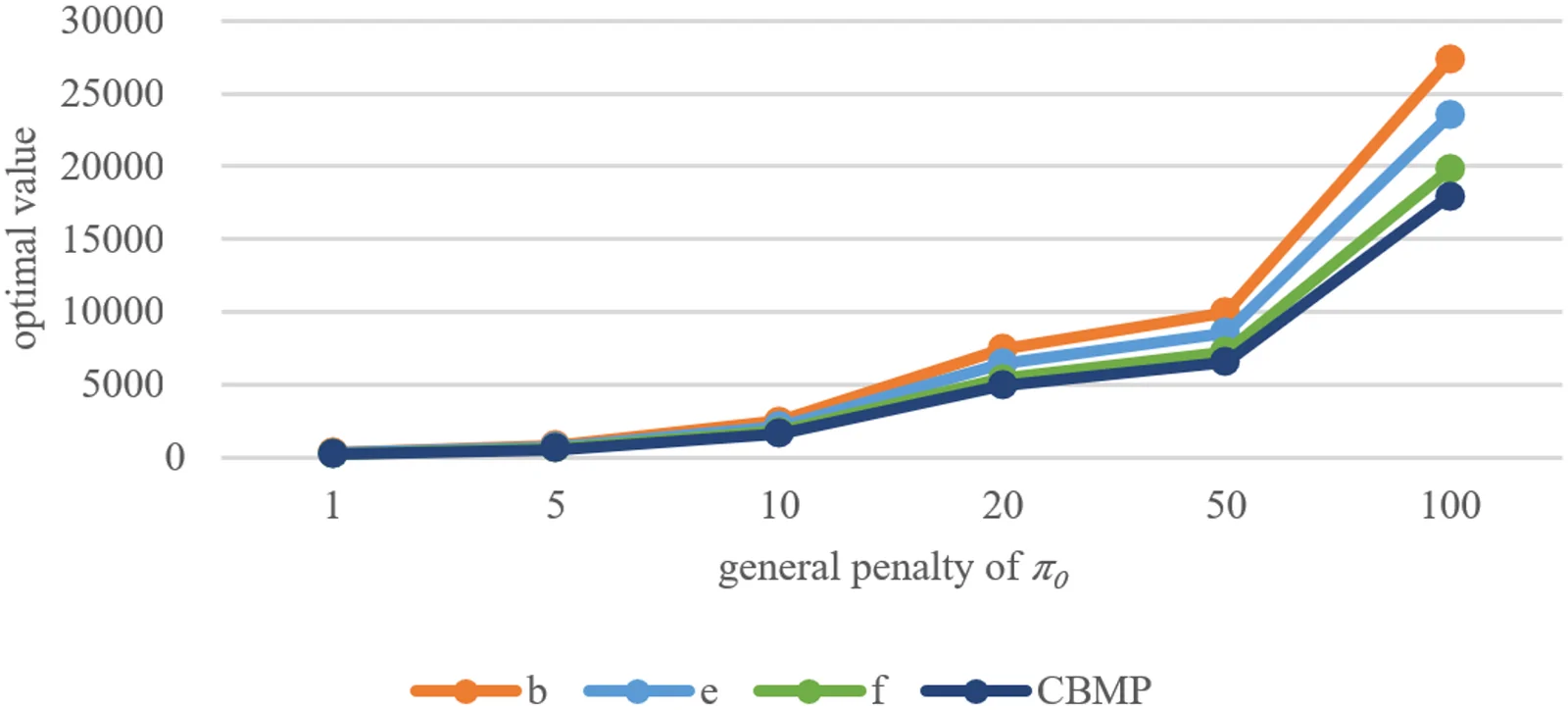
The effect of penalty of π_0_ on the optimal value under different policies (static and dynamic).

**Fig 5:**
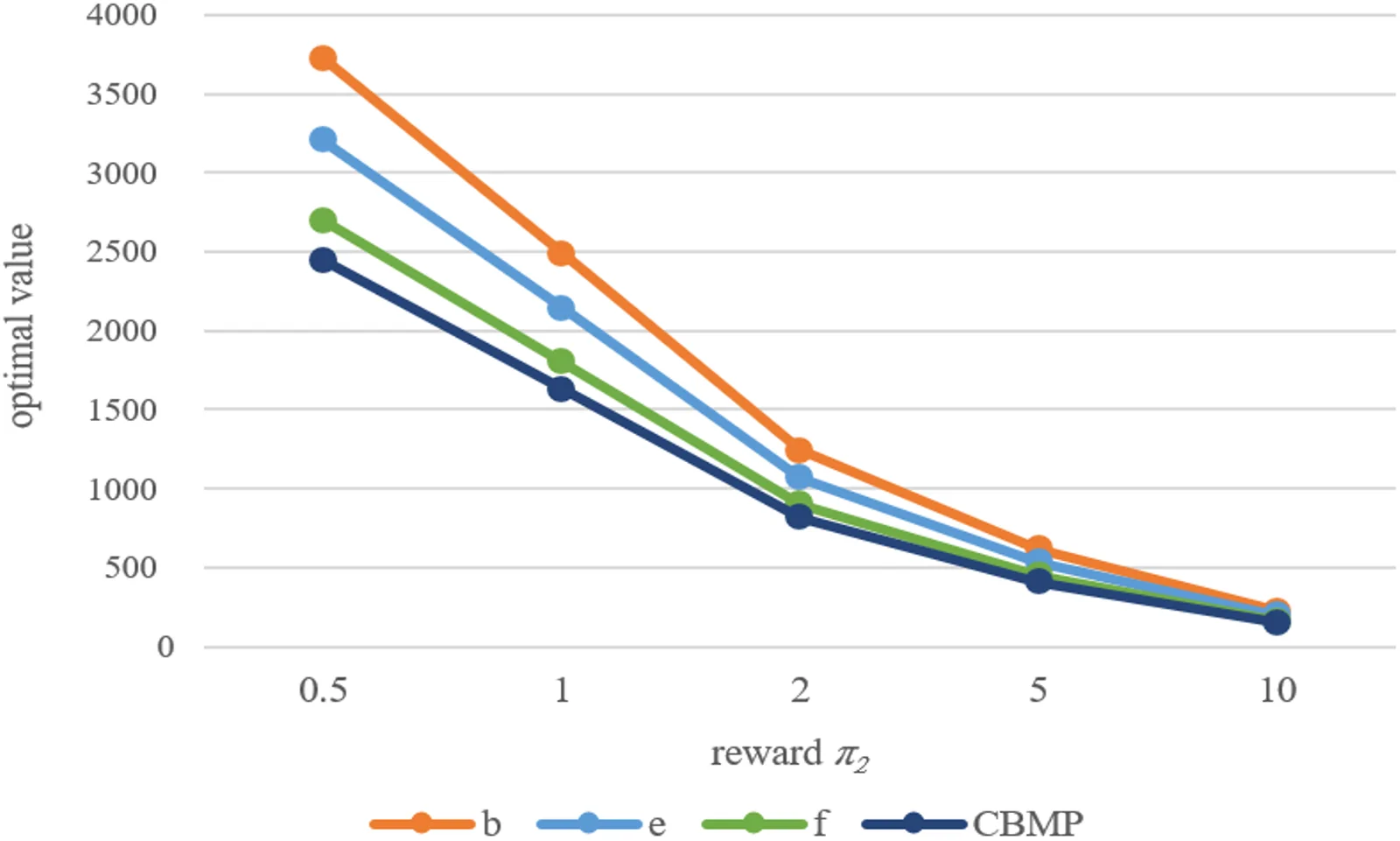
The effect of reward *π*_*2*_ on the optimal value under different policies (static and dynamic).

**Fig 6:**
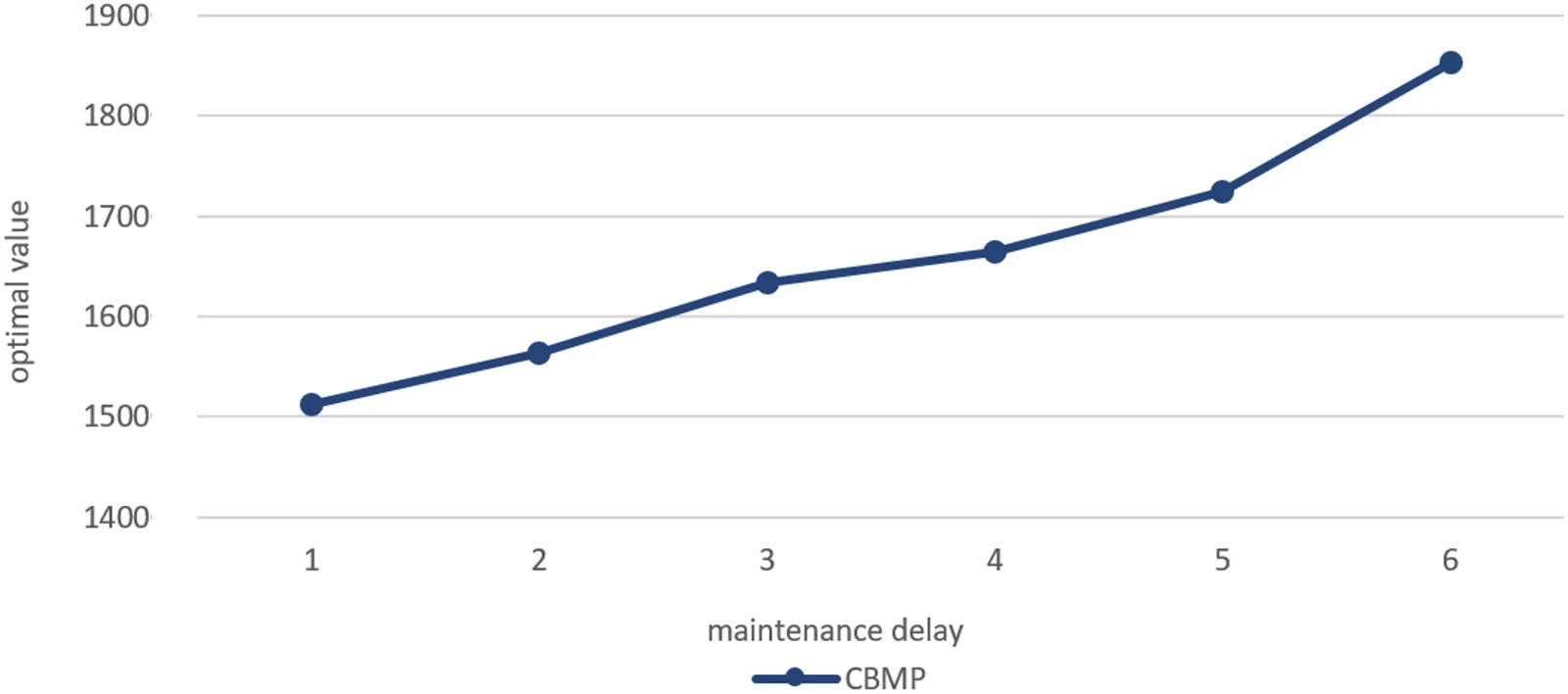
The effect of maintenance delay on the optimal value in CBMP policy.

Next, for different parameters of corrective maintenance cost, penalty $\pi_{\text{1}}$, reward $\pi_{\text{2}}$, and maintenance delay, the optimal value for the initial state, i.e., the expected total costs of the planning horizon, is obtained, and the trend of changes for policies b, e, f and CBMP is shown in Figs 3–6.

In Fig 3, the optimal value of the system for different levels of corrective maintenance cost ($C_{CM}$) and for four different policies including three fixed policies (b, e, f) and one dynamic policy (CBMP) is shown. As the corrective maintenance cost increases, the optimal value increases for all policies. This increase occurs with a relatively steep slope, indicating the direct and significant effect of corrective maintenance costs on the overall system performance. The dynamic CBMP policy performs better than the other policies at all cost levels and produces a lower optimal value. The difference between CBMP and other policies becomes more noticeable at high costs (e.g., $C_{CM}=$10,000), indicating that the dynamic policy has a better ability to handle costly conditions.

Fig 4 shows the initial state optimal value for policies b, e, f and CBMP for different parameters $\pi_{\text{0}}$, which is the penalty (assuming that other problem parameters are fixed).

Fig 5 shows the initial state optimal value for policies b, e, f and CBMP for different parameters $\pi_{\text{2}}$, which is the reward (assuming that other problem parameters are fixed). As the reward value ($\pi_{\text{2}}$) increases, the optimal value decreases, because increasing the production reward compensates for the total costs.

Fig 6 shows the impact of maintenance delay on the total system cost within the CBMP policy. As illustrated, longer maintenance delays lead to higher overall costs. This effect stems from the fact that when the delay increases, the time required to initiate maintenance actions becomes longer. In such circumstances, the only feasible measure to mitigate further deterioration is to reduce the production rate, which in turn results in an inability to fully satisfy demand and thereby increases system costs. Conversely, operating the system at a higher production rate accelerates deterioration and leads to earlier failures. Under long maintenance delays, this situation can cause complete system downtime before maintenance can be performed, resulting simultaneously in unmet demand costs and higher maintenance costs due to more severe failures. These findings highlight a critical trade-off between production rate, demand satisfaction, and maintenance planning, emphasizing the necessity of minimizing maintenance delays to achieve cost efficiency and system reliability.

In the following, the presented problem will be compared with the case where there is no maintenance delay. In the case, where the maintenance delay is zero, maintenance operations do not require scheduling time, and the state of the problem will be defined by two parameters: the system failure status and the number of the current period in the planning horizon. Therefore, the MDP state will be defined as (i, t).

Similar to the base model of Section 4, for this problem, it is assumed that conducting PM/CM actions requires one period, although the scheduling time is zero. In addition, the recursive functions have also undergone slight changes compared to the equations described in Section 4, which are presented as Equations 12–14.


$$\begin{array}{cc} V(i,\;t)=\; & Min\{\underset{k\in K}{\text{min}}\left\{{\pi_{\text{0}}.y_{t}+\pi }_{\text{1}}.max\left\{\text{0},\varphi_{t}-Z_{t}\;\right\}-\pi_{\text{2}}.max\left\{\text{0},Z_{t}-\varphi_{t}\;\right\}+\sum_{i=j}^{m}P_{ij}^{K}\;V(j,\;t+\text{1})\right\},\;\\ & {A+C}_{pm}+\pi_{\text{0}}{+\;\pi }_{\text{1}}*\;\varphi_{t}+\;V(\text{0},\;t+\text{1})\} \end{array}$$ (12)

$$\begin{array}{c} \forall i\text{≠}m;t\epsilon \left\{\text{1},\;\text{2},\;\text{3},\;\ldots ,T\right\} \end{array}$$

$$\begin{array}{c} V(m,\;t)=Min\;\left\{A+C_{cm}+\pi_{\text{0}}{+\;\pi }_{\text{1}}*\;\varphi_{t}+\;V(\text{0},\;t+\text{1})\;,\;\pi_{\text{0}}{+\;\pi }_{\text{1}}*\;\varphi_{t}+\;V(m,\;t+\text{1})\right\}\;\forall \;t\;\epsilon \;\left\{\text{2},\;\text{3},\;\ldots ,T\right\} \end{array}$$ (13)

$$\begin{array}{c} V(i,\;T+\text{1})=\left\{ \begin{array}{c} @lC_{cm}\;i=m\\ C_{pm}\;\text{0}<i<m\\ \text{0}\;i=\text{0} \end{array}\right. \end{array}$$ (14)

In this case, the problem is solved for the parameters presented in Section 5 using the value iteration algorithm, and the optimal policy and optimal value-function of each state are presented in Tables 41 and 42, respectively. In Tables 40 and 41, the horizontal rows indicate the period (t={1, 2,…, 10,11}), and the vertical rows indicate the system’s failure status (i={0, 1, 2, 3}). Accordingly, the optimal expected cost during the planning horizon is 1447.6 which is the state value function for (0,ψ,1).

**Table 41:** Optimal policy for each state when maintenance delay is zero.

τ	i										
	1	2	3	4	5	6	7	8	9	10	11
0	4	4	0	4	4	1	4	1	3	4	Do nothing
1	–	3	0	4	2	PM	2	PM	PM	4	PM
2	–	4	PM	4	PM	PM	4	PM	PM	4	PM
3	–	Do nothing	CM	CM	CM	CM	Do nothing	CM	CM	Do nothing	CM

**Table 42:** Optimal value for each state when maintenance delay is zero.

τ	i										
	1	2	3	4	5	6	7	8	9	10	11
0	1447.6	825.76	787.26	756.26	506.51	501.25	452	433.5	378.5	215	0
1	–	888.26	812.26	829.74	567	538	512.5	458.5	496	279	0
2	–	953.26	812.26	894.25	602.25	542	579.5	458.5	529	342	0
3	–	1203.3	992.26	1132.5	782.25	718	789.5	638.5	676	651	0

Subsequently, for the case when the maintenance delay is zero, the optimal values of the initial state for the six constant policies are obtained by the policy iteration algorithm and presented in Table 43. The initial state is equal to the state in which the system is at the beginning of the planning horizon (t=1) and the failure status of the system is in the as-good-as-new state (i=0), i.e., the system state is: (i,t)=(0, 1).

**Table 43:** The operational costs of the system during the planning horizon under dynamic CBMP policy and 6 constant policies when maintenance delay is zero.

Policy	a	b	c	d	e	f	CBMP
Optimal value	2014	2313.2	1821.5	1905.4	1780	1601.7	1447.6

According to Table 43, as when the system has a maintenance delay, CBMP dynamic policy leads to lowest expected operational costs during the planning horizon. The CBMP policy adjusts the decisions of production rates and maintenance scheduling according to the conditions of the system and consequently decreases the operational costs of the system. Comparing expected costs under CBMP policy for the case when δ=0 with the case that δ=3 reveals that maintenance delay increases the expected costs during the planning horizon which it seems reasonable.

## 8. Conclusion

In production management and engineering, manufacturers always want to maximize their profits by meeting customer demand and managing costs. Production planning is necessary to increase revenue, profit, and customer satisfaction. Due to the complexity and characteristics of large-scale equipment, their deterioration and failure become inevitable. Today, with the presence of the Internet of Things and data mining techniques, managers can improve operational decisions by adjusting the production process and creating dynamic maintenance and repair plans. In this research, a single-component system with fixed and predetermined demand is considered, and the production rate is related to the system failure rate. The presented problem, when there is a maintenance delay, is modeled in the form of a Markov decision process, and then it is adopted by the value iteration algorithm of optimal policies. In this article, a practical example is presented to show the application of the problem. In addition, the dynamic CBMP policy is compared with other fixed policies such as the fixed maintenance policies and the fixed production policies. The results show that the CBMP policy is an optimal policy because it makes decisions dynamically and according to the system conditions in each state. Furthermore, the problem presented for the case where there is no maintenance delay is also modeled, and the output results of the dynamic CBMP policy are compared with other presented fixed policies. In this case as well, the dynamic CBMP policy leads to better results. Finally, the results of the algorithm have been reviewed and analyzed. As future research, this problem can be extended to a system with two and multi components, or the demand can be considered as random. Extending the model to multi-unit systems would also require explicitly modeling limited maintenance-resource (technician) capacity, since multiple machines may require maintenance concurrently and compete for the same repair resources — a consideration that does not arise in the single-unit setting studied here. Another limitation of the present study is the assumption that surplus production is sold immediately without inventory holding costs; incorporating an inventory-holding mechanism to capture make-to-stock environments is a valuable direction for future research.
